# Smoothness metrics for reaching performance after stroke. Part 1: which one to choose?

**DOI:** 10.1186/s12984-021-00949-6

**Published:** 2021-10-26

**Authors:** Mohamed Irfan Mohamed Refai, Mique Saes, Bouke L. Scheltinga, Joost van Kordelaar, Johannes B. J. Bussmann, Peter H. Veltink, Jaap H. Buurke, Carel G. M. Meskers, Erwin E. H. van Wegen, Gert Kwakkel, Bert-Jan F. van Beijnum

**Affiliations:** 1 Department of Biomedical Signals and Systems, Technical Medical Centre, University of Twente, Enschede, The Netherlands; 2 Department of Rehabilitation Medicine, Amsterdam Movement Sciences, Amsterdam Neuroscience, Amsterdam UMC, Vrije Universiteit Amsterdam, de Boelelaan 1117, Amsterdam, The Netherlands; 3 Department of Rehabilitation Medicine, Erasmus MC, University Medical Centre Rotterdam, Rotterdam, The Netherlands; 4 Rehabilitation Technology, Roessingh Research and Development, Enschede, The Netherlands; 5 Department of Physical Therapy and Human Movement Sciences, Feinberg School of Medicine, Northwestern University, Chicago, Il USA; 6 Department of Neurorehabilitation, Amsterdam Rehabilitation Research Centre, Reade, Amsterdam, The Netherlands

**Keywords:** Stroke, Reaching, Smoothness, Review, Simulation analyses

## Abstract

**Background:**

Smoothness is commonly used for measuring movement quality of the upper paretic limb during reaching tasks after stroke. Many different smoothness metrics have been used in stroke research, but a ‘valid’ metric has not been identified. A systematic review and subsequent rigorous analysis of smoothness metrics used in stroke research, in terms of their mathematical definitions and response to simulated perturbations, is needed to conclude whether they are valid for measuring smoothness. Our objective was to provide a recommendation for metrics that reflect smoothness after stroke based on: (1) a systematic review of smoothness metrics for reaching used in stroke research, (2) the mathematical description of the metrics, and (3) the response of metrics to simulated changes associated with smoothness deficits in the reaching profile.

**Methods:**

The systematic review was performed by screening electronic databases using combined keyword groups *Stroke*, *Reaching* and *Smoothness*. Subsequently, each metric identified was assessed with mathematical criteria regarding smoothness: (a) being dimensionless, (b) being reproducible, (c) being based on rate of change of position, and (d) not being a linear transform of other smoothness metrics. The resulting metrics were tested for their response to simulated changes in reaching using models of velocity profiles with varying reaching distances and durations, harmonic disturbances, noise, and sub-movements. Two reaching tasks were simulated; reach-to-point and reach-to-grasp. The metrics that responded as expected in all simulation analyses were considered to be valid.

**Results:**

The systematic review identified 32 different smoothness metrics, 17 of which were excluded based on mathematical criteria, and 13 more as they did not respond as expected in all simulation analyses. Eventually, we found that, for reach-to-point and reach-to-grasp movements, only *Spectral Arc Length* (SPARC) was found to be a valid metric.

**Conclusions:**

Based on this systematic review and simulation analyses, we recommend the use of SPARC as a valid smoothness metric in both reach-to-point and reach-to-grasp tasks of the upper limb after stroke. However, further research is needed to understand the time course of smoothness measured with SPARC for the upper limb early post stroke, preferably in longitudinal studies.

**Supplementary Information:**

The online version contains supplementary material available at 10.1186/s12984-021-00949-6.

## Introduction

Stroke is one of the main causes of adult disability [7–9]. Goal-directed upper limb movements after stroke are characterized by slowness, spatial and temporal discontinuity (i.e., lack of smoothness), and abnormal stereotypic patterns of muscle activation or movement synergies [10, 11].

Currently, stroke literature offers several ways for objective measurement of upper limb movement, and standardization is lacking [12, 13]. Measuring changes in smoothness during reaching, pointing or grasping using the upper paretic limb is suggested to reflect quality of movement (QoM) early after stroke [5, 6]. Smoothness of movement is regarded as the result of ‘learned, coordinative processes in sensorimotor control’, although the underlying neuronal and mechanical substrates that cause lack of smoothness in motor control are still poorly understood [14, 15]. Smoothness is therefore interpreted as a reflection of the level of sensorimotor coordination and movement proficiency [16, 17].

Balasubramanian and colleagues defined *movement smoothness* as continuity or non-intermittency of a movement, independent of its amplitude and duration [6]. Maximizing the smoothness of a movement is considered to be prioritized by the neuro-muscular system, as it reduces the control burden on the brain [18]. Nonetheless, the neurophysiological mechanisms of smoothness deficits after stroke are yet to be understood. Muscle activity patterns observed during reaching after stroke have been shown to be impaired [19]. Smoothness deficits could, for example, be caused by the inability to synchronize motor units or control agonists and antagonists in the right proportions [14, 20], or may be due to changes in cortico-spinal tract excitability following stroke [21].

A prerequisite for investigating smoothness deficits after stroke is identifying a ‘valid’ smoothness metric. Unfortunately, there is currently no commonly accepted metric for quantifying movement smoothness, and many types have been used in the literature to investigate smoothness of reaching movements post stroke [13]. The use of many smoothness metrics in clinical research is limited by several methodological concerns. For instance, some metrics are not clearly described and therefore not reproducible. Other metrics depend on the duration or distance of reaching or are not dimensionless. In both cases, they could be confounded by the shape, *i.e.*, the duration and amplitude, of the movement [16]. Some proposed smoothness metrics are based on position, and do not truly reflect smoothness per se [6, 22] as they do not measure the rate of change of position. Furthermore, some metrics are linear transformations of other smoothness metrics, and are therefore proxies of existing metrics. Finally, some metrics lack robustness against measurement noise [6].

Several narrative reviews about smoothness have discussed the strengths and weaknesses of a limited set of available metrics [6, 13, 14, 16]. The relations between these metrics and smoothness were assessed either by using simulation models, or by studying post-stroke correlations with clinical scales. However, these studies reviewed the literature narratively, rather than systematically. Therefore, a comprehensive overview of metrics used to measure smoothness after stroke is lacking. Furthermore, these metrics have not been validated in terms of whether they reflect smoothness [23]. As a result, proper recommendations for a valid smoothness metric are currently lacking in the literature.

Our goal was to identify the most valid metrics for quantifying smoothness of upper paretic limb movement after stroke during reaching tasks [24]. Reaching can be used to extend or point the hand/arms (reach-to-point) or touch or grasp something (reach-to-grasp). To this end, several subsidiary questions were formulated. Firstly, to identify available metrics, we addressed the question ‘*Which metrics have been used in the literature to assess movement smoothness in reaching by persons with stroke?*’. Secondly, we filtered metrics sequentially, using a set of criteria derived from the literature to assess whether their mathematical definitions regarding smoothness were sound [6, 14, 16]. This was done to answer the question ‘*Which of the available metrics are mathematically defined, reproducible, not linear transforms of another metric, dimensionless, and defined using the rate of change in position?*’. Thirdly, we assessed how each metric responds to smoothness deficits in the reaching task, to answer the question ‘*How does each smoothness metric respond to a simulated change in the velocity profile of a reaching task?’*. In this study, metrics that satisfy the two latter questions can be said to be valid smoothness metrics that have been applied in stroke research.

## Materials and methods

### Systematic literature review

The literature search was performed in accordance with the PRISMA statement, using keyword groups ‘Stroke’, ‘Reaching’ and ‘Smoothness’ [25] (Full search query in Additional file 1.A). PubMed, Scopus, Cochrane Library, EMBASE and CINAHL databases were searched for all records up to October 2019. The screening of the literature was performed by one author (BLS) and ambiguities were resolved with another author (MRMI). Articles were excluded if they were in a language other than English, or if they were reviews. Eventually, we included articles in which (1) reaching or aiming movements of persons with stroke were studied and (2) a metric was used to determine the smoothness of a reaching movement. The International Classification of Functioning, Disability, and Health (ICF) definition of a *reaching* movement (code: d4452) is ‘*Using the hands and arms to extend outwards and touch and grasp something, such as when reaching across a table or desk for a book*’ [26]. The references of the included articles were scanned for additional suitable articles. The review has been registered in the PROSPERO registry under CRD42020173211.

### Metrics mathematically reflecting smoothness

Metrics should reflect the definition of movement smoothness, i.e., the continuity or non-intermittency of the movement profile, independent of its amplitude and duration [6]. Additionally, as smoothness reflects continuity, it should be based on rate of change of position or a higher derivative. Based on the requirements stated in the introduction above, the definition of a metric was not sound if:

1. the metric was not dimensionless,
2. the metric was not reproducible from the literature,
3. the metric was not based on velocity or a derivative of velocity, or
4. the metric was linearly related to another metric by (a) scaling or (b) addition of a constant.

### Response of metrics to changes in velocity profile

The response of each metric to four different types of simulated perturbations, applied to two reaching velocity profiles, viz. reach-to-point and reach-to-grasp, were studied. A reach-to-point movement was simulated using a minimal jerk model [27]:

1 $$v_{{{\text{mj}}}} \left( t \right) = {\text{d}}_{{\text{t}}} \left( {\frac{{30t^{4} }}{{T^{5} }} - \frac{{60t^{3} }}{{T^{4} }} + \frac{{30t^{2} }}{{T^{3} }}} \right)$$

where *v*_*mj*_ is the minimal jerk velocity profile, ${\mathrm{d}}_{\mathrm{t}}$ is the total reaching distance, *T* is the total movement time and *t* is the time scale from 0 to *T*. Using this, a symmetrical velocity profile (v_symm_) was created with a ${\mathrm{d}}_{\mathrm{t}}$ of 0.3 m, and a *T* of 1 s. While this velocity profile reflects a reach-to-point movement, it does not truly reflect reach-to-grasp movements [28], as the latter movements have to account for a higher accuracy when nearing the target position [28]. An initial analysis on healthy subjects showed that an asymmetrical velocity profile (v_asymm_) was better suited for this purpose. This was modelled using a polynomial curve (Additional file 1.B). Both velocity profiles are shown in Additional file 1.C, and have been further investigated.

Of the four simulated perturbations, the first three are analytical evaluations of the smoothness metrics, and the last one is specifically based on theories regarding recovery of movement after stroke [14].

- *Shape Simulation (SS)* The movement duration and distance of the base velocity profiles were varied. The smoothness metric must not depend on either of these parameters.

The durations and distances of both velocity profiles were varied from 0.5 to 6.0 s in steps of 0.1 s, and from 0.2 to 0.7 m in steps of 0.01 m. A total of 2856 combinations were used to calculate the outcomes of the metrics. The ranges for movement duration and distance were chosen such that they were within the physiological range of human reaching.

- *Harmonic Disturbances (HD)* In this analysis, tremor or weak control of reaching movement was simulated using harmonic disturbances added to the base velocity profiles [29]. This included sinusoids with varying amplitude and frequency. The relation between frequency or amplitude and the metric should be monotonic. Smoothness is expected to decrease with increasing amplitude for a given frequency, and also with increasing frequency for a given amplitude.

Sinusoids of frequencies between 2 and 25 Hz in steps of 0.5 Hz, and amplitudes between 0 and 0.2 m/s in steps of 0.005 m/s were added to the base velocity profile. A total of 1927 unique combinations were explored. The ranges chosen were within the physiological ranges of movement [4, 30].

- *Measurement noise (MN)* A more robust smoothness metric is less sensitive to measurement noise [6]. The noise was modelled as normally distributed white noise (mean = 0, standard deviation = 1) and added to the base velocity profiles.

The root mean square (RMS) of the noise was varied from 0 to 0.08 m/s in steps of 0.002 m/s. Twenty-five different realizations for each RMS were generated, and the metrics were estimated for each realization. The minimum, maximum, mean and standard deviation of the metrics were calculated and reported. In an additional analysis of noise we filtered the noise-added velocity profile using a zero phase 4th order low pass Butterworth filter with cut off of 20 Hz [6]. The mean of the metric outcome across the 25 realizations after filtering was determined.

- *Sub-movement Simulation (SMS)* A smoothness metric must reflect movement intermittency, and the change in the progressive blending of sub-movements [6, 31]. The smoothness metric should therefore decrease monotonically with increasing number of sub-movements and increasing delays between each sub-movement.

This is an extension of previous work applied to a set of metrics [4, 14]. The reaching profiles were modelled as a composition of two or more sub-movements, each defined as the base velocity profile with a duration of 1 s. The sub-movements were separated by a varying lag, denoted as *Ks*. *Ks* ranged from 0 s, were the sub-movements fully overlap, to 1.2 s, where there was 1.2 s between the starting points of the two sub-movements. The lag was increased in steps of 0.02 s. Note that when the lag was greater than 1 s, there were instances of zero velocity between subsequent sub-movements. The total duration of the movement increased with *Ks*. Simulations were performed for 2–4 sub-movements.

#### Analysis of the simulations

The responses of each metric to the four different simulated perturbations were individually assessed. For the *Shape Simulation* and *Harmonic Disturbances,* the percentage change (%Δ) of the metric from its value estimated using the respective base profile was identified as

$$\%\Delta = \frac{metri{c}_{i} -metri{c}_{1}}{metri{c}_{1}} *100$$

where $metri{c}_{i}$ corresponds to metric values for each combination of parameters in the simulations, and $metri{c}_{1}$ is the value for the first combination used. For *Shape Simulation*, $metri{c}_{1}$ corresponded to the smoothness of a base profile with reaching distance 0.2 m and duration 0.5 s. We considered a change of more than 10% as meaningful, and the maximum %Δ was identified.

For *Harmonic Disturbances*, $metri{c}_{1}$ corresponded to a base profile of reaching distance 0.3 m and duration 1 s. The %Δ was estimated for each combination of frequency and amplitude. Then, a Combinations Exceeded (CE) parameter was marked as the percentage of the combinations that exceeded 10%. A higher value of CE meant that there were more combinations of frequency and amplitude that caused a meaningful change in the value of the metric from its base velocity profile.

For the *Measurement Noise* simulation, the ratio of signal-to-noise power (SNR) was estimated to quantify the robustness to noise. First, the power of the measurement noise was estimated. Then, the power of the signal was estimated as the power of the base velocity profile with added measurement noise. The lowest RMS of added noise was 0.002 m/s, which corresponds to SNRs of 45.0 dB for v_symm_ and 45.4 dB for v_asymm_. Subsequently, the highest noise RMS added was 0.08 m/s, which corresponded to SNRs of 13.2 dB for v_symm_ and 13.6 dB for v_asymm_. The SNR at which the mean value of the metric differed from the base velocity profile by at least 10% is reported. Metrics that reached a 10% threshold only at a high RMS of added measurement noise, and therefore a low SNR, were deemed to be more robust to noise. On the other hand, metrics that crossed the threshold at lower RMS values, and therefore a higher SNR, were highly sensitive to noise. An SNR threshold to distinguish between high and low robustness was determined using the distribution of the SNR values obtained at the 10% cut-off for each metric. Metrics with an SNR lower than the 25th percentile were considered to have *high* robustness to noise, and all others were deemed to have *low* robustness to noise.

Finally, in the *Sub-movements Simulations*, the change in the direction of the derivative of the metrics for increasing delays was assessed to study monotonicity. All computations were performed using MATLAB (2018b, The Mathworks, Natick, MA, USA).

### Data availability

The MATLAB scripts used to generate the different simulations, the scripts for estimating the smoothness metrics, and the resulting metrics are provided with this manuscript (Additional file 4).

## Results

### Systematic literature review

**Fig. 1:**
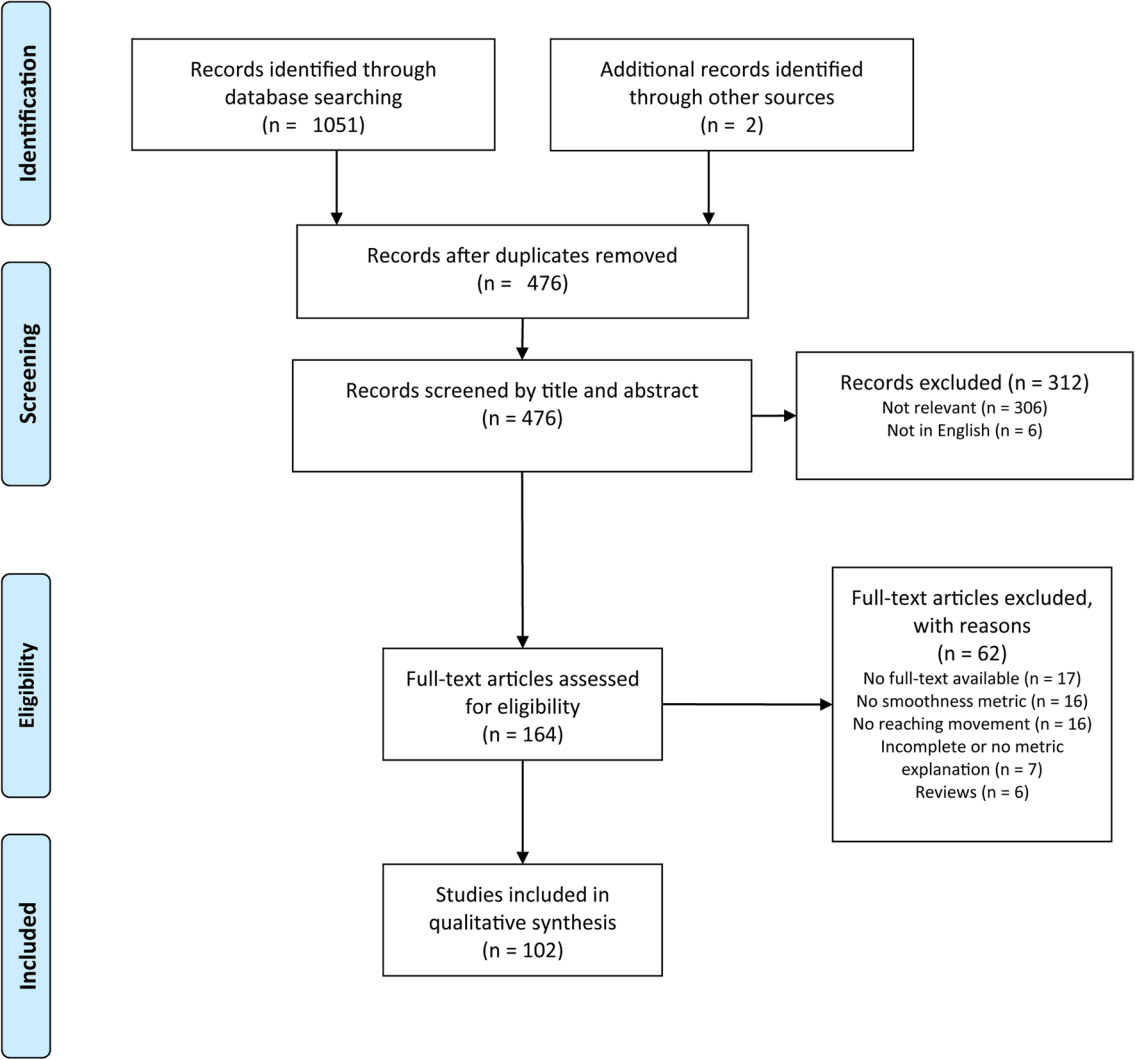
PRISMA flow chart

A total of 476 unique articles were identified, 102 of which were found to be eligible for inclusion using Rayyan [32]. A total of 32 different metrics (Additional file 1.D, E) were identified. Figure 1 shows the PRISMA flow chart (Additional file 3 reports the PRISMA checklist).

### Metrics mathematically reflecting smoothness

Table 1 shows an overview of all metrics identified from the literature, and the ones that did not meet the four exclusion criteria (E1–E4). The metrics identified in the systematic review were classified into categories based on their mathematical definitions. Metrics defined in the time domain were classified as ‘Trajectory metrics’, or ‘Velocity metrics’, or ‘Acceleration metrics’, or ‘Jerk metrics’. Metrics defined in the frequency domain were classified as ‘Frequency metrics’. Metrics that did not fit in any of these categories, or fitted in more than one category, were classified as ‘Other metrics’.

**Table 1:** Overview of Smoothness metrics identified from the literature

Metric (Abbreviation)	Units	Articles used^a^	Exclusions	Category	Earliest citation
Index of curvature (IC)	[]	1	E3	Trajectory	[33]
Standard deviation in 2D plane (SD_XY)	[]	1	E3	Trajectory	[72]
Number of sub-movements (NOS)	[]	1		Velocity	[37]
Speed metric (SM)	[]	15		Velocity	[14]
Normalized reaching speed (NRS)	[]	2	E4 (*SM*)	Velocity	[35]
Movement arrest period ratio (MAPR)	[]	3		Velocity	[34]
Tent Metric (TM)	[]	1	E2	Velocity	[14]
Velocity Arc Length (VAL)	[]	1		Velocity	[4]
Correlation Metric (CM)	[]	2		Velocity	[38]
Peaks Metric (Peaks)	[]	61		Acceleration	[39]
Number of Movement Units (NMU)	[]	3	E4a (*Peaks*)	Acceleration	[73]
Number of peaks normalized by movement duration (NPt)	[s^−1^]	1	E1	Acceleration	[40]
Number of peaks normalized by movement distance (NPd)	[m^−1^]	3	E1	Acceleration	[41]
Inverse number of peaks and valleys (IPV)	[]	1		Acceleration	[44]
Acceleration metric (AM)	[]	2	E1	Acceleration	[35]
Integrated absolute jerk (IAJ)	[ms^−2^]	2	E1	Jerk	[74]
Mean absolute jerk (MAJ)	[ms^−3^]	2	E1	Jerk	[33]
Mean absolute jerk normalized by peak speed (MAJPS)	[s^−2^]	7	E1	Jerk	[14]
Integrated squared jerk (ISJ)	[m^2^s^−5^]	1	E1	Jerk	[75]
Root mean squared jerk metric (RMSJ)	[ms^−3^]	1	E1	Jerk	[76]
Normalized integrated jerk (NIJ)	[ms^−3^ $\sqrt{\mathrm{s}}$]	1	E1	Jerk	[77]
Dimensionless squared jerk (DSJt)	[]	12		Jerk	[3]
Log dimensionless squared jerk (LDSJt)	[]	1		Jerk	[5]
Dimensionless squared jerk (DSJm)	[]	1	E4a (*DSJt*)	Jerk	[2]
Dimensionless squared jerk (DSJb)	[]	1		Jerk	[4]
Log dimensionless squared jerk (LDSJb)	[]	1		Jerk	[4]
Rotational jerk (RJ)	[]	1	E3	Jerk	[48]
Spectral metric (SPMR)	[]	1		Frequency	[49]
Spectral method (SPM)	[]	1		Frequency	[1]
Spectral arc length 2012 (SPAL)	[]	8		Frequency	[4]
Spectral arc length (SPARC)	[]	1		Frequency	[6]
Combined smoothness metric (CSM)	[− −]^b^	1	E1	Other	[78]

^*a*^Number of articles in the systematic review that used the metric. ^b^Units were not available. Exclusion criteria include E1: metric was not dimensionless; E2: metric not reproducible from the literature; E3: metric not based on velocity or its derivative; and E4: metric linearly related to another metric (shown in brackets) by (a) scaling or (b) addition of a constant

*Trajectory-based smoothness metrics﻿*: The Index of Curvature *(IC)* [33] and the standard deviation of the position perpendicular to the movement direction *(SD_XY)* measured smoothness using only the discrete position information of the reaching movement. As these are not based on the rate of change of position as a function of time, they cannot be used to measure continuity and thereby smoothness of reaching (criterion E3). This holds for any proposed metric that belongs to this category.

*Velocity-based smoothness metrics*: Of the seven velocity-based metrics, Movement Arrest Period Ratio (*MAPR*), Speed Metric (*SM*), Number of Sub-movements (*NOS*), Velocity Arc Length (*VAL*) and Correlation Metric (*CM*) were found to be mathematically sound for measuring smoothness and were used for further analysis.

*MAPR* is the proportion of time that the movement speed exceeds a given percentage of the peak speed [34]. *SM*, defined as the mean speed of the whole movement normalized by the peak speed, was found to decrease with the severity of the stroke [14]. Normalized Reaching Speed (*NRS*) is the ratio of the difference in peak and mean speed over the peak speed [35]. As *NRS* = 1 − *SM*, it is a linear transform of the *SM* metric, and is expected to behave congruently. Therefore, *NRS* was excluded from further analysis (criterion E4). The definition and mathematical description of the Tent Metric (*TM*) was incomplete in the study [14], and therefore could not be evaluated further (criterion E2). *NOS* counts the sub-movements that make up the norm of the velocity profile [36] and has been used to assess smoothness in persons with stroke [37]. *VAL* [4] is based on the arc length of the speed profile normalized by the peak speed. It assumes that a bell-shaped velocity profile has a shorter arc length than one with velocity fluctuations. *CM* determines the correlation between the velocity profile extracted from the minimal jerk model and the actual hand velocity profile during reaching [38].

*Acceleration-based smoothness metrics*: In this category, six metrics were identified, of which peaks (*Peaks*) and Inverse Number of Peaks and Valleys (*IPV*) were analysed further.

*Peaks* was the most frequently used metric (61 citations). The metric reflects the number of local maxima in the velocity profile for a given movement [39], which is inversely proportional to the smoothness of a movement. *Peaks* can also be defined as zero crossings in the acceleration domain when the derivative of the acceleration is negative. *Peaks* were additionally normalized either to the movement duration (*NPt*) [40] or to the movement distance (*NPd*) [41]. However, doing so causes the metric to be dependent on movement duration or movement distance. Therefore, these adapted definitions of *Peaks* (*NPt* and *NPd*) were excluded (criterion E1). Smoothness was also estimated using the Number of Valleys [42] or the Number of Valleys and Peaks [43]. Since these definitions are linear transforms of *Peaks*, they are assumed to show congruent behaviour to *Peaks*, and were excluded from further analysis (criterion E4). *IPV*, on the other hand, is not a linear transform of *Peaks*, and was included in further analysis [44]. Although a few studies employed additional criteria for peak detection [45, 46], the choices for these criteria, and the difference with *Peaks* was not explicitly provided, and they were not considered for the present study. The Acceleration Metric (*AM*) is the ratio between the mean acceleration and the peak acceleration [35]. A point-to-point reaching movement should have zero velocity both at the beginning and end of the movement, which implies that the mean acceleration over this movement must be zero. However, this was not the case in the referenced studies, suggesting that some aspect of its definition is missing [35, 47]. According to the textual description, the metric definition is not face-valid, and it was therefore excluded (criterion E2).

*Jerk-based smoothness metrics*: There were a total of 12 different jerk-based metrics, of which only two types of dimensionless squared jerk metrics, *DSJt* and *DSJb,* and their respective log transformations, *LDSJt*, and *LDSJb*, were further analysed.

Jerk, the third derivative of position, has often been used as a measure of smoothness in different ways; either as the integral of the squared jerk or the integral of the absolute jerk [3, 14, 16]. Furthermore, the results were scaled using different terms, which introduces a unit to the metric. As smoothness metrics have to be dimensionless (criterion E1), only the dimensionless jerk metrics were considered. Three types of dimensionless squared jerk metrics, *DSJt* [3]*, DSJb* [4]*,* and *DSJm* [2]*,* were introduced to measure smoothness. The suffixed letter corresponds to the author’s name. These jerk metrics differ in the normalizations used in their definitions. As *DSJm* is a linear transform of *DSJt*, it was excluded (criterion E4a). A natural logarithm transform of the *DSJb* metric was performed to improve its sensitivity (*LDSJb*) [4]. The same was applied to *DSJt*, thereby introducing *LDSJt* [5]. As *LDSJb* and *LDSJt* employ the peak velocity, and the average velocity respectively in their equations, they are not linear transformations of each other. Rotational Jerk (*RJ*) measures movement smoothness using the orientations of the wrist during the movement [48]. This form of smoothness quantifies the variability of hand orientation. However, as we analysed changes to a tangential velocity profile, we have no models for the changes in orientation during the reaching movement. Therefore, this metric was not analysed further.

*Frequency-based smoothness metrics*: All four metrics from this category, including Spectral Method (*SPM*), Spectral Arc Length 2012 (*SPAL*), Spectral Arc Length (*SPARC*)*,* and Spectral Metric (*SPMR*), were analysed further.

The *SPM*, *SPAL*, and *SPARC* were developed by the same authors [1, 4, 6], and are directly proportional to the increase in smoothness of the movement. The *SPM* measures smoothness as the sum of all peaks in the amplitude-normalized Fourier transform of the velocity profile [1]. The *SPAL* uses the negative arc length of the amplitude and the frequency-normalized Fourier transform of the velocity profile [4]. The frequency range used in *SPAL* was further limited in order to define *SPARC* [6]. Finally, *SPMR* expresses smoothness using the energy within a 0.2 Hz bin around the dominant frequency in the Fourier transform of the accelerations, normalized by the entire energy [49].

*Other metrics*: Kostić and Popović [50] defined a smoothness metric (Combined Smoothness Metric [*CSM*]) in the context of a drawing task in which a patient, while seated at a desk, draws a pre-defined square. The smoothness metric uses information from the movement velocity and jerk, and consists of four different terms. As the formula uses different dimensions incorrectly, the metric was excluded (criterion E1).

### Response of metrics to changes in velocity profile

In the previous section, fifteen metrics were identified as mathematically sound, and therefore subjected to further analysis: *NOS*, *SM*, *MAPR*, *VAL*, *Peaks*, *IPV*, *DSJt*, *LDSJt*, *DSJb*, *LDSJb*, *CM*, *SPMR*, *SPM*, *SPAL* and *SPARC*. Table 2 describes the selected metrics’ range of feasible mathematical values obtained for each type of perturbation. The parameters used to interpret the response of metrics to the simulations (%Δ, CE, and SNR) are also shown. Metrics *SM, MAPR, IPV, CM*, *SPM*, *SPMR*, *SPAL* and *SPARC* should decrease with decreasing smoothness of movement. However, the other metrics increase with decreasing smoothness. To enable comparison across metrics, we append a * to these latter metrics. This includes NOS*, VAL*, Peaks*, DSJt*, LDSJt*, DSJb*, and LDSJb*.

**Table 2:** Simulation Analysis for each metric and its changes

Metric (Feasible Range)	Base Velocity Profile	Shape			Sinus			Noise			Filtered noise			Sub-movements (N = 2)	
		Min	Max	%Δ (%)	Min	Max	CE (%)	Min	Max	SNR (dB)	Min	Max	SNR (dB)	Min	Max
NOS* (1–7)	v_symm_	1	2	50	1	7	10.7	1	7	N.A	1	7	N.A	1	3
	v_asymm_	3	7	N.A	3	7	1.2	4	7	N.A	4	7	N.A	3	7
SM (0–1)	v_symm_	0.5	0.5	0	0.4	0.6	63.6	0.4	0.5	18.6	0.4	0.6	–	0.5	0.7
	v_asymm_	0. 5	0.5	0	0.4	0.5	56	0.3	0.5	18.6	0.4	0.5	–	0.4	0.5
MAPR (0–1)	v_symm_	0.8	0.8	0	0.8	1	3.2	0.8	0.9	–	0.8	0.9	–	0.7	0.9
	v_asymm_	0.8	0.8	0	0.7	0.9	0.6	0.7	0.9	–	0.7	0.9	–	0.7	0.9
VAL* (− ∞–∞)	v_symm_	− 2E − 04	− 1.6E − 05	91.7	− 1E− 03	− 5.7E − 04	21.6	− 9.7E − 03	5.6E − 03	25	− 9.7E − 03	− 7.2E − 03	19	− 4.9E − 03	− 2E − 03
	v_asymm_	− 2E − 04	− 1.7E − 05	91.7	− 1E − 03	− 6.6E − 04	16.2	− 9.7E − 03	4.2E − 03	24.6	− 9.7E − 03	− 7.9E − 03	18.2	− 4.9E − 03	− 2E − 03
Peaks* (1–∞)	v_symm_	1	1	0	1	25	92.2	1	36	45	1	16	43.3	1	3
	v_asymm_	1	1	0	1	25	93.4	1	35	45.4	1	16	43.7	1	2
IPV (− ∞–1)	v_symm_	1	1	0	0	1	92.2	0	1	45	0	1	43.3	0.2	1
	v_asymm_	1	1	0	0	1	93.4	0	1	45.4	0	1	43.7	0.3	1
DSJt* (0–∞)	v_symm_	19	19	0	17.4	8.2E + 3	96.3	18.7	5.2E + 03	45	18.5	885.6	49.3	18.8	95.6
	v_asymm_	36.4	36.7	0.1	34.4	8.2E + 3	94	34.4	5.2E + 3	45.4	33.3	889.8	49.7	34.6	179.6
LDSJt* (0–∞)	v_symm_	2.9	2.9	0	2.9	9	95.1	2.9	8.6	45	2.9	6.8	49.3	2.9	4.6
	v_asymm_	3.6	3.6	0	4	9	90.9	3.5	8.6	45.4	3.5	6.8	43.7	3.5	5.2
DSJb* (0–∞)	v_symm_	204.6	204.8	0.1	162.5	2.1E + 07	96.9	194.8	9.9E + 06	45	191.4	3.8E + 05	49.3	199.7	4.3E + 03
	v_asymm_	548	549.3	0.3	489.1	1.6E + 07	95	478.6	8.5E + 06	45.4	457	2.7E + 05	49.7	437	1.1E + 04
LDSJb* (0–∞)	v_symm_	5.3	5.3	0	5.1	16.9	95.1	5.3	16.1	45	5.3	12.9	49.3	5.3	8.4
	v_asymm_	6.3	6.3	0	6.2	16.6	91.6	6.2	16	45.4	6.1	12.5	46.7	6.1	9.3
CM (− 1–1)	v_symm_	1	1	0	0.8	1	34.5	0.9	1	–	1	1	–	− 0.2	1
	v_asymm_	0.6	0.6	0.1	0.4	0.6	26.7	0.6	0.7	–	0.6	0.7	–	− 0.1	0.8
SPMR (0–1)	v_symm_	0.1	1	774.4	0.1	0.2	93.5	0	0.2	39	0	0.2	35.3	0.2	0.5
	v_asymm_	0.1	0.9	1E + 03	0.1	0.2	76.9	0	0.2	39.4	0	0.2	32.8	0.1	0.3
SPM (0–∞)	v_symm_	− 1	− 1	0	− 1.4	− 1	57	− 2.1	− 1	27	− 1.6	− 1	25.8	− 1.8	− 1
	v_asymm_	− 1	− 1	0	− 1.4	− 1	55	− 2.1	− 1	27.4	− 1.6	− 1	26.8	− 2	− 1
SPAL (0–∞)	v_symm_	− 2.1	− 1.9	11	− 3	− 2	50	− 2.3	− 2	–	− 2.3	− 2	–	− 3.4	− 1.9
	v_asymm_	− 2	− 1.8	12.2	− 3	− 1.8	54.7	− 2.2	− 1.9	–	− 2.2	− 1.8	–	− 3.8	− 1.9
SPARC (0–∞)	v_symm_	− 1.4	− 1.4	1	− 2.9	− 1.4	66.9	− 2.2	− 1.4	15.1	− 2.2	− 1.4	19	− 2.7	− 1.4
	v_asymm_	− 1.4	− 1.4	0.4	− 2.8	− 1.4	66.3	− 2.1	− 1.4	14.7	− 2.1	− 1.4	18.2	− 3.1	− 1.4

Assessing the response of each metric by comparing the effect of perturbation against the base velocity profile: Δ%: percentage difference in metric value from the base velocity profiles (instances where the metric depends on the shape are in bold), CE(%): percentage of combinations where the metric value differs by at least 10% from base velocity profiles, SNR(dB): the signal-to-noise ratio at which the metric differs by at least 10% from the base profile. Note that a higher added RMS noise value corresponds to a lower SNR value, and hence to a greater robustness to noise. Metrics included are NOS* (number of sub-movements), *SM* (speed metric), *MAPR* (movement arrest period ratio), VAL* (velocity arc length), Peaks* (number of peaks), *IPV* (inverse of number of peaks and valleys), DSJt* and DSJb* (Dimensionless squared jerk), LDSJb* and LDSJt* (log of DSJt* and DSJb*), *CM* (correlation metric), *SPMR* (spectral metric), *SPM* (spectral method), *SPAL* (spectral arc length 2012), and *SPARC* (spectral arc length)

In this section, we discuss the results of the simulation analyses using v_symm_ as the base velocity profile. As the changes in the values of the smoothness metrics for the v_asymm_ were similar, their results have been placed in Additional file 1.F. The main difference between using the two base velocity profiles was the magnitude of the resulting values, as shown in Table 2. Where other differences in the response to the simulation analyses were found, they are addressed in the following sections.

#### Shape simulation (SS)

**Fig. 2:**
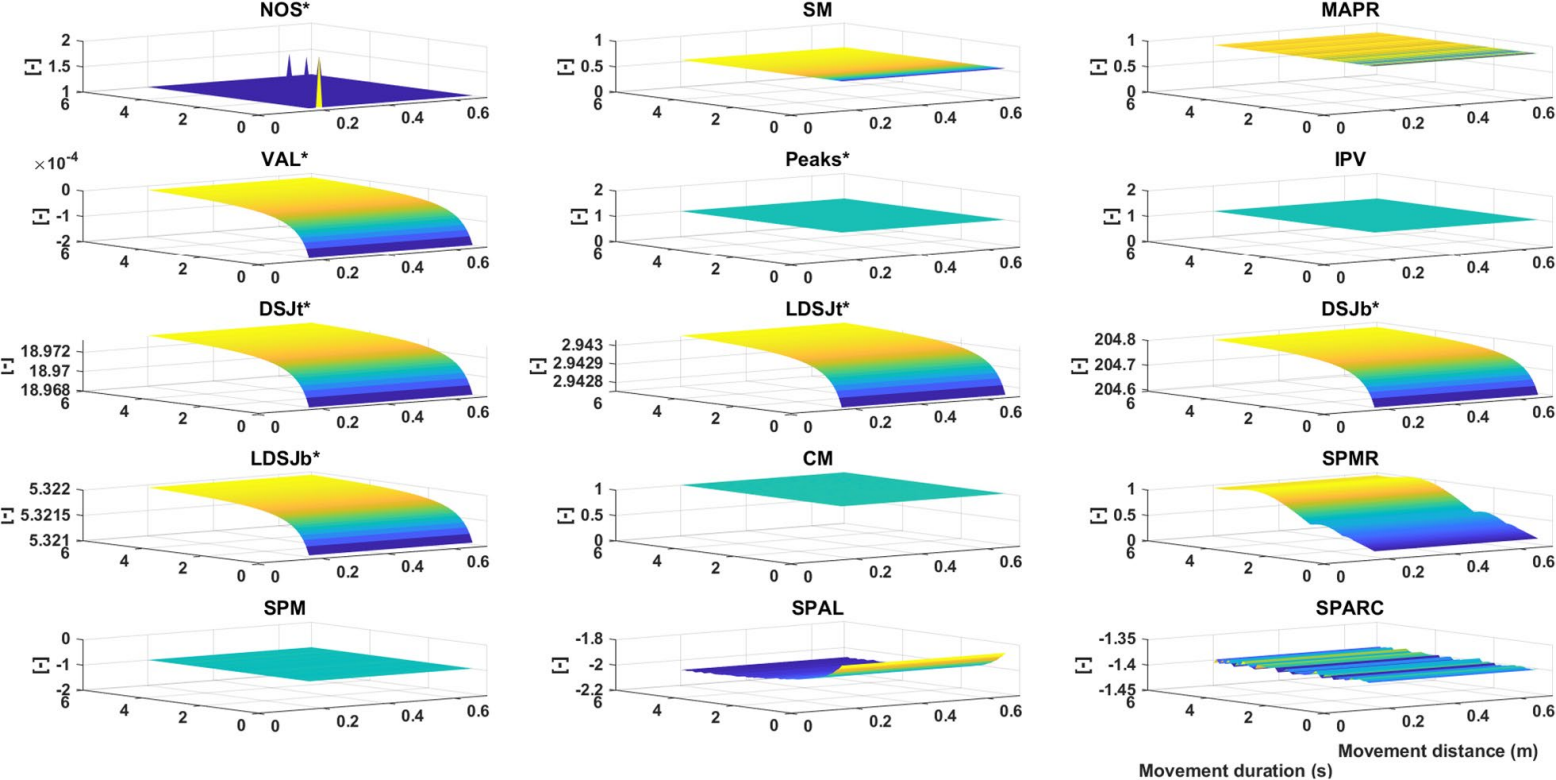
Shape simulation. The vertical axis represents the metric value decreasing from yellow to blue. The horizontal axes represent the movement duration and movement distance. Metrics included are NOS* (number of sub-movements), *SM* (speed metric), *MAPR* (movement arrest period ratio), VAL* (velocity arc length), Peaks* (number of peaks), *IPV* (inverse of number of peaks and valleys), DSJt* and DSJb* (Dimensionless squared jerk), LDSJb* and LDSJt* (log of DSJt* and DSJb*), *CM* (correlation metric), *SPMR* (spectral metric), *SPM* (spectral method), *SPAL* (spectral arc length 2012), and *SPARC* (spectral arc length). By definition, the metrics with a * increase with decreasing smoothness

Figure 2 shows the response of each metric to changes in movement duration and movement distance for the symmetric velocity profile. The percentage of change (%Δ) shows that NOS*, VAL*, *SPAL*, and *SPMR* were sensitive to changes in this simulation for both velocity profiles (Table 2). The inconsistencies in the number of sub-movements as measured by the NOS* shows that this metric is not suitable as a smoothness metric. Metrics *SM*, *MAPR*, Peaks*, *IPV*, LDSJt*, LDSJb*, *CM*, and *SPM* were truly insensitive to changes in this simulation.

#### Harmonic disturbances (HD)

**Fig. 3:**
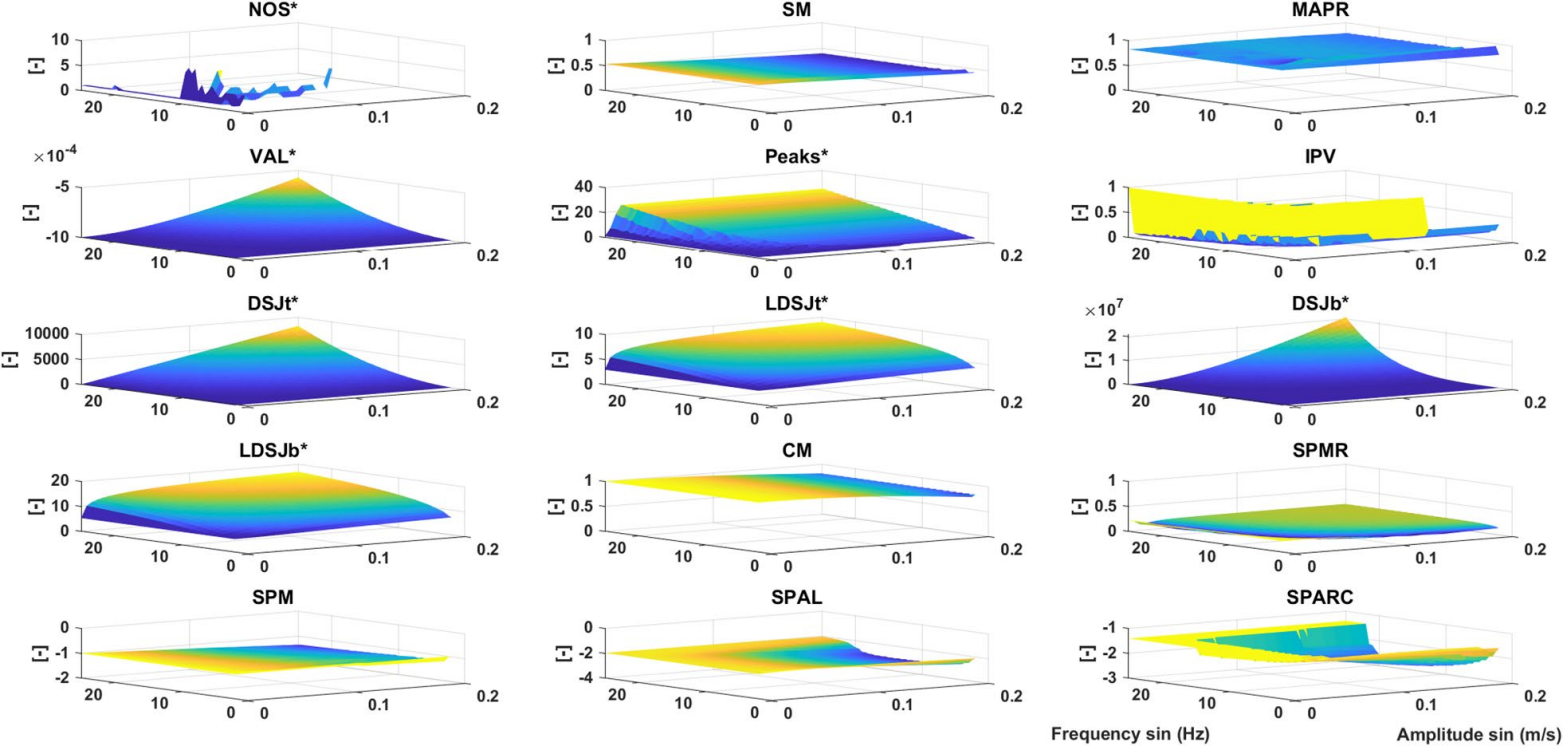
Harmonic Disturbances. The vertical axis represents the metric value decreasing from yellow to blue. Metrics included are NOS* (number of sub-movements), *SM* (speed metric), *MAPR* (movement arrest period ratio), VAL* (velocity arc length), Peaks* (number of peaks), *IPV* (inverse of number of peaks and valleys), DSJt* and DSJb* (Dimensionless squared jerk), LDSJb* and LDSJt* (log of DSJt* and DSJb*), *CM* (correlation metric), *SPMR* (spectral metric), *SPM* (spectral method), *SPAL* (spectral arc length 2012), and *SPARC* (spectral arc length). By definition, the metrics with a * increase with decreasing smoothness

Figure 3 shows the metric outcomes with added sines of varying frequencies and amplitudes. The algorithm used to estimate NOS* failed to converge to an optimal solution for higher frequencies (missing data in Fig. 3). All other metrics behave as expected to this simulation and show a lower smoothness outcome as the amplitude of the added sine increases. However, all metrics except *SM*, *MAPR* and *CM* showed lower smoothness outcomes at higher frequencies for the same amplitude. *SPAL* and *SPARC* were insensitive to sine disturbances with frequencies higher than 20 Hz, as their definitions include the use of a cut-off frequency. The CE values for NOS*, *MAPR*, VAL*, and *CM* are less than 50% (Table 2) suggesting that these metrics are relatively less sensitive to harmonic disturbances, and might not be useful to reflect presence of tremor or weak control of reaching movement.

#### Measurement noise (MN)

**Fig. 4:**
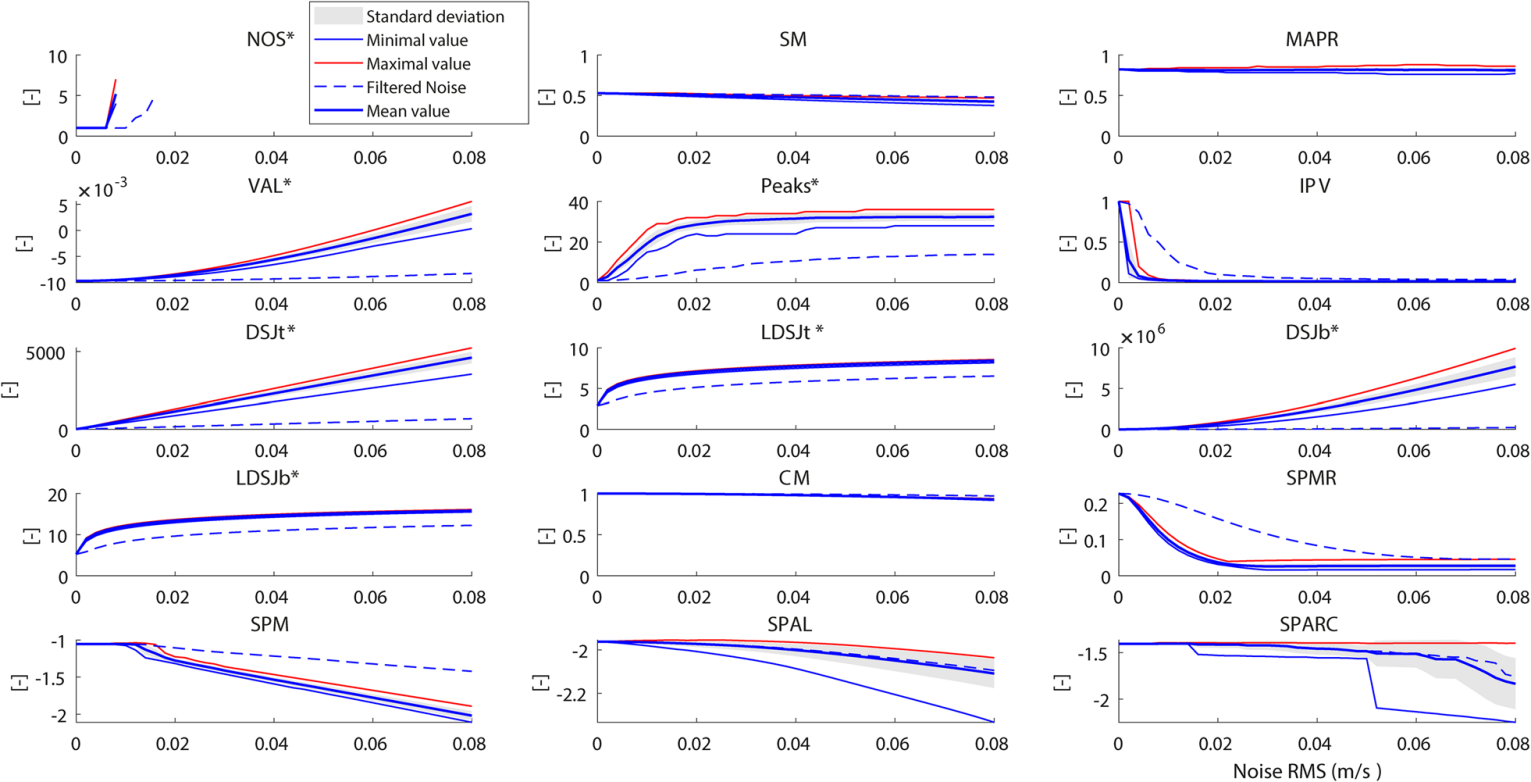
Measurement Noise. The thick blue line represents the mean value of 25 different realizations of the noise for each measurement noise level added, and the shaded area is the corresponding standard deviation. The dotted black lines denote the minimum and maximum values of the metric found at that RMS value. The dashed blue line shows mean values of the filtered noise sets. Metrics included are NOS* (number of sub-movements), *SM* (speed metric), *MAPR* (movement arrest period ratio), VAL* (velocity arc length), Peaks* (number of peaks), *IPV* (inverse of number of peaks and valleys), DSJt* and DSJb* (Dimensionless squared jerk), LDSJb* and LDSJt* (log of DSJt* and DSJb*), *CM* (correlation metric), *SPMR* (spectral metric), *SPM* (spectral method), *SPAL* (spectral arc length 2012), and *SPARC* (spectral arc length). By definition, the metrics with a * increase with decreasing smoothness

NOS* is only capable of analysing the smoothness at low noise powers up to an RMS of 0.008 m/s (Fig. 4). For higher noise powers, the algorithm that counts NOS* fails to converge to an optimal solution (indicated by N.A. in Table 2 in the SNR column). The other metrics show lower outcomes of smoothness as the RMS of the noise is increased (Fig. 4). *MAPR*, *CM*, and *SPAL* did not cross the 10% threshold for any noise power included in the simulation (unfilled entries ‘–’ in Table 2). This indicates that these metrics are robust to the range of measurement noises added in this study. Peaks*, *IPV,* and all jerk-based smoothness metrics were very sensitive to measurement noise.

#### Sub-movements simulation (SMS)

**Fig. 5:**
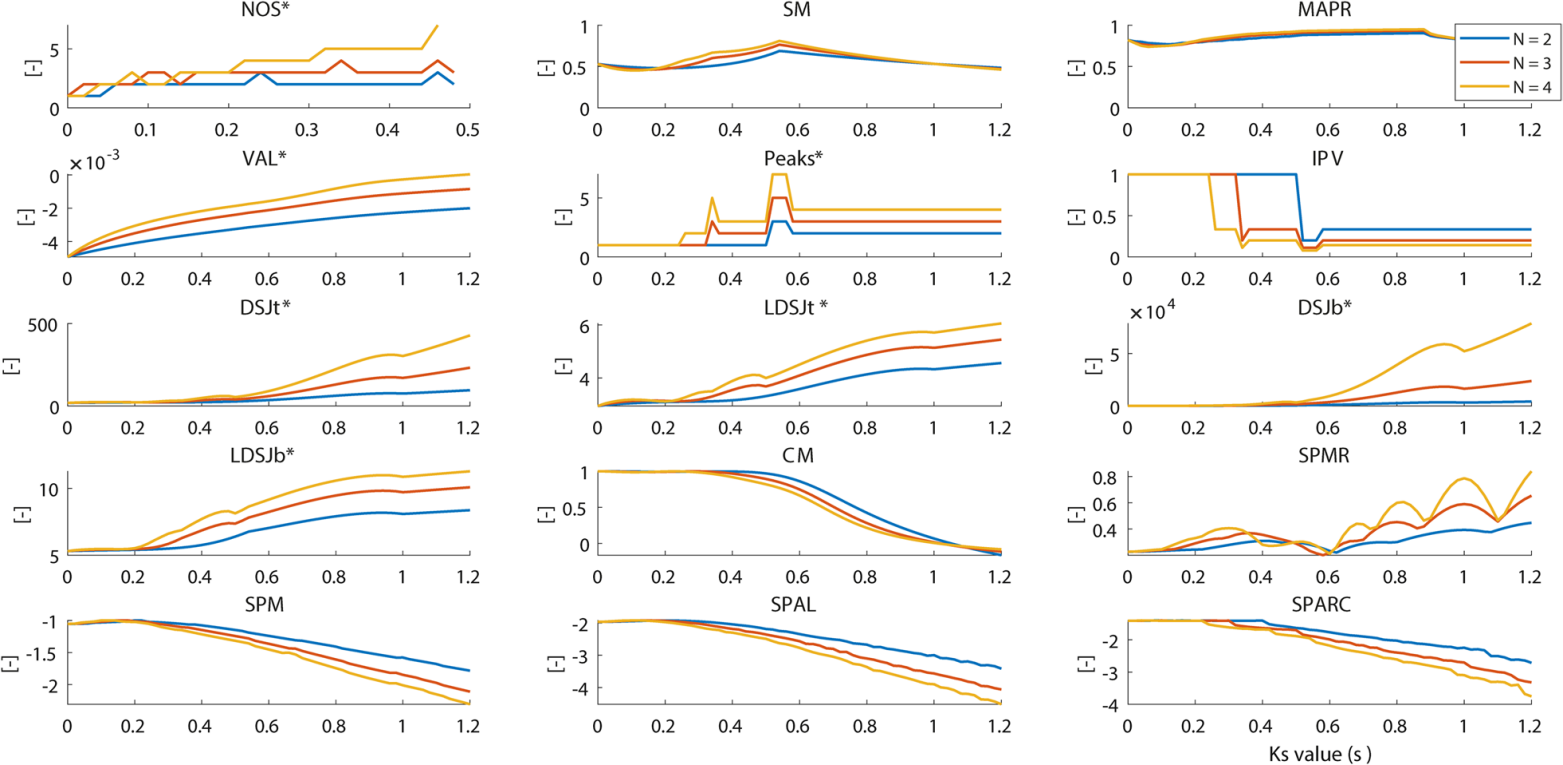
Sub-movements simulation. The colours denote the number of sub-movements. The horizontal axis represents the lag between two sub-movements. Metrics included are NOS* (number of sub-movements), *SM* (speed metric), *MAPR* (movement arrest period ratio), VAL* (velocity arc length), Peaks* (number of peaks), *IPV* (inverse of number of peaks and valleys), DSJt* and DSJb* (Dimensionless squared jerk), LDSJb* and LDSJt* (log of DSJt* and DSJb*), *CM* (correlation metric), *SPMR* (spectral metric), *SPM* (spectral method), *SPAL* (spectral arc length 2012), and *SPARC* (spectral arc length). By definition, the metrics with a * increase with decreasing smoothness

The algorithm used to estimate NOS* calculated incorrect values at certain instances (Fig. 5). This was because the algorithm did not converge to an optimal solution within the provided boundary constraints with increasing number of sub-movements. We found that only the VAL* was truly monotonic to changes in lag between sub-movements (Additional file 1.G). *SPMR* surprisingly increased with increasing numbers of sub-movements which shows that the metric fails in this analysis. All other metrics showed a lower outcome for smoothness with increasing number of sub-movements and increasing delay between them. For Peaks* and *IPV*, a third peak was detected at 0.3 and 0.5 s (Fig. 5). Although non monotonic overall, the metrics Peaks*, *IPV*, *SPM*, *SPAL*, and *SPARC* showed jumps only at certain discrete intervals. The *CM* was seen to be monotonic only if the delay between sub-movements was larger than 0.2 s. Further, when considering increases in delays (*Ks*) of 0.06 s, the *SPAL* and *SPARC* metrics also showed a monotonic change for delays larger than 0.2 s. Furthermore, the monotonicity was influenced by the base velocity profile used for all metrics except VAL*, *SPMR*, and *SPARC* (Additional file 1.G).

#### Summary of findings

Table 3 summarizes the simulation analysis results and indicates whether the responses of each metric were as expected. For the measurement noise analysis, the robustness of each metric to added noise was studied. Descriptive statistics of the SNR values as shown in Table 2 were used to divide the metrics into two groups; *high* and *low* robustness to measurement noise. Note that a higher added RMS noise value corresponds to a lower SNR value, and hence to greater robustness to noise. We find that only *SPARC* responded as expected to the *Shape Simulation*, *Harmonic Disturbances*, and *Measurement Noise* simulations. For the *Sub-movement Simulation*, *SPARC* responded as expected by showing a monotonic change for increase in delays between sub-movements greater than 0.2 s (20% of sub-movement duration) only when the delay was increased in steps of 0.06 s (6% of sub-movement duration).

**Table 3:** Summary of the analysis results

Metric	Duration/distance independence		Harmonic disturbances		Sub-movements		Robustness	
	v_symm_	v_asymm_	v_symm_	v_asymm_	v_symm_	v_asymm_	v_symm_	v_asymm_
NOS*	No	No	No	No	No	No	No data^+^	
SM	Yes^1^	Yes^1^	Yes	Yes	No	No	High	High^4^
MAPR	Yes^1^	Yes^1^	No	No	No	No	High^4^	High^4^
VAL*	No	No	No	No	Yes	Yes	High	High
Peaks*	Yes^1^	Yes^1^	Yes	Yes	No	No	Low	Low
IPV	Yes^1^	Yes^1^	Yes	Yes	No	No	Low	Low
DSJt*	Yes^1^	Yes	Yes	Yes	No	No	Low	Low
LDSJt*	Yes^1^	Yes^1^	Yes	Yes	No	No	Low	Low
DSJb*	Yes	Yes	Yes	Yes	No	No	Low	Low
LDSJb*	Yes^1^	Yes^1^	Yes	Yes	No	No	Low	Low
CM	Yes^1^	Yes	No	No	No^2^	No^2^	High^4^	High^4^
SPMR	No	No	Yes	Yes	No	No	Low	Low
SPM	Yes^1^	Yes^1^	Yes	Yes	No	No	Low	Low
SPAL	No	No	Yes	Yes	No^2,3^	No^2,3^	High^4^	High^4^
SPARC	Yes	Yes	Yes	Yes	No^2,3^	No^2,3^	High	High

‘Yes’ means that the metric responded to the perturbations as expected, whereas ‘No’ means otherwise. ^1^There was no instance in the analysis where the metric value crossed the 10% threshold. ^2^The metric showed monotonic change for lag values greater than 0.2 s. ^3^The metric showed monotonic change when the derivative was estimated using steps of 0.06 s for the lag between sub-movements. ^4^ The metric was robust to all noise values added in the simulation. ^+^Incomplete data. Metrics included are NOS* (number of sub-movements), *SM* (speed metric), *MAPR* (movement arrest period ratio), VAL* (velocity arc length), Peaks* (number of peaks), *IPV* (inverse of number of peaks and valleys), DSJt* and DSJb* (Dimensionless squared jerk), LDSJb* and LDSJt* (log of DSJt* and DSJb*), *CM* (correlation metric), *SPMR* (spectral metric), *SPM* (spectral method), *SPAL* (spectral arc length 2012), and *SPARC* (spectral arc length)

## Discussion

The aim of this study was to identify valid smoothness metrics to investigate the QoM of the upper paretic limb during reaching tasks by persons with stroke. A smoothness metric used in stroke research was valid if it was mathematically sound, and responded to the simulation analyses as expected. The systematic literature review revealed 32 different metrics used in stroke research, however, only 15 unique metrics had a sound mathematical definition relating to smoothness [16]. Many metrics were sensitive to reaching distance and duration, or were not found to be useful to reflect presence of tremor or weak control of reaching movement, or were not robust to added measurement noise. We find that almost all metrics do not change monotonically to increasing delay between the sub-movements. Further, we observe in some cases (Table 3) that the reaching task influences the behaviour of smoothness metric, which was a disadvantage to certain metrics. Our simulation analyses showed that Spectral Arc Length (*SPARC*) responded favourably in all simulation analyses, for both base velocity profiles, and therefore is a valid metric to measure smoothness of reach-to-point or reach-to-grasp movements post stroke.

The simulation analyses performed in this study builds on and agrees with the trends for the shape, noise, and sub-movement simulations shown in literature [4, 6, 14, 16]. However, this study offers an exhaustive analysis of all available smoothness measures and also offers insight on influence of added sinusoids.

### Clinical relevance

Smoothness is considered a result of learned coordinative processes, and increased motor control results in improved smoothness during reaching, pointing and grasping [5, 6, 14]. Identifying and using valid smoothness metrics is essential for proper clinical research, and results in accurate observations of the recovery of motor control while improving the identification of true treatment effects on QoM. The present study showed that only *SPARC* is a valid smoothness metrics in spite of the plethora available in the literature.

Neurological recovery occurs spontaneously after stroke and results in normalization of neurological measures such as EEG patterns, whereas behavioural restitution is rather restricted to regaining normal behaviour, not denying that neuronal restitution is taking place [51, 52]. Clinical assessments which are most closely related to behavioural restitution and thereby neurological recovery, take into account the ability to perform movements outside the pathologic synergies [53]. Whether smoothness metrics reflect neurological recovery after stroke can be determined by investigating the longitudinal association between clinical outcomes that measure behavioural restitution and smoothness metrics [54]. Furthermore, studying the associations between the recovery of neurological pathways and changes in movement smoothness will reveal the influence of behavioural restitution and compensation on smoothness. Additionally, identifying neurological recovery along with changes in movement smoothness post stroke and eventually the underlying physiology that governs smoothness, will provide an indication whether smoothness can be used as a target or outcome measure in training and in designing rehabilitation robotics. In these cases, smoothness measured during reaching in healthy age- and gender-matched individuals can be used as reference values [54].

This study used simulations to offer a systematic analysis of changes to the reaching profiles. In case of harmonic disturbance analysis, the upper limit of the sinusoidal frequency range tested (25 Hz) was beyond known frequencies in stroke, and therefore covers all potential disturbances [55]. In case of noise simulation analysis, the robustness of metrics to added measurement noise was tested. However, if the noise is a result of weak human control, the resulting movement would be less smooth, as reflected by the smoothness metric. Therefore, efforts to distinguish between measurement noise and perturbations due to actual human motion control must be undertaken in order to distinguish abnormal, pathologically reduced movement smoothness from that seen in healthy, age- and gender-matched subjects.

### Practical barriers

In order to measure smoothness, the measurement system should be capable of measuring velocity (or a higher derivative) of reaching. Measuring smoothness using motion tracking systems or high-end kinematic measurement sensors is relatively simple using the *SPARC* metric. However, practical requirements need to be considered when the metric is applied in either a clinical setting or an ambulatory or daily life setting. For ambulatory or daily life settings, metrics that can be estimated using wearable on-body sensors are preferred. Inertial and Magnetic Measurement Units (IMUs) are commonly used as wearable sensors for measuring the kinematics of movement. However, as an IMU measures accelerations, estimating velocity from it would require additional processing and is usually prone to drift [56]. In this study, we measured *SPARC* using linear velocities [6]. Alternatively, in a recent study, Melendez-Calderon and colleagues suggest that during reaching, *SPARC* can be measured using angular velocities obtained from IMUs [22]. However, techniques to correct drift due to strapdown integration [56] were not employed in their study, as the authors suggest that it warrants a systematic analysis of the errors introduced in the smoothness estimate [22]. Therefore, if the errors are accounted for, it should be possible to reliably measure *SPARC* using corrected linear velocities obtained from IMUs for a standardized pre-defined movement with a clear start and end posture. Given the advantages of using IMUs, their validity in measuring QoM after stroke requires further research [57].

### Generalizability of current findings

Besides stroke, smoothness is highly relevant for studying the impact of neurological disease in other populations, such as those with Parkinson’s and Huntington’s disease [16]. For instance, smoothness has been used to study *fluidity* of movement in the upper limb, reflecting bradykinesia and rigidity in patients with Parkinson’s disease [58]. Furthermore, the generalizability of smoothness should be investigated for the lower limb allowing to differentiate between affected and healthy gait, as well as to examine effects of medication on smoothness, and to identify fall risk [59]. In addition, the level of smoothness is highly relevant in sports as a measure of proficiency [60, 61]. The present findings may serve as inspiration for related fields to determine how smoothness varies for the movement task they analyse.

### Limitations and future directions

The first limitation of the current review was that it was restricted to smoothness metrics investigated in post-stroke reaching. Additional metrics for measuring movement smoothness could have been identified if our review was not limited to stroke studies. Generalization to other neurological diseases is therefore limited. The same is true for other movement tasks such as rhythmic drinking tasks [62] or self-paced, isolated elbow flexion movements [63]. Secondly, only English language articles were considered for our systematic review.

Thirdly, we model different reaching tasks with different velocity profiles; reach-to-point or aiming movements with symmetrical velocity profiles based on minimum jerk models [27], and reach-to-grasp movement with an asymmetrical velocity profile based on a polynomial curve [28]. The minimum jerk profile was shown to be a good approximation for reaching in healthy individuals [14, 64–69]. The asymmetric profile was modelled by applying a polynomial fit to reach-to-grasp movements in healthy individuals using a polynomial fit. This fit was found to be better than averaging the reaching profiles from the healthy individuals (Additional file 1.B). However, a true measure of smoothness should not be influenced by the movement profile.

Fourthly, the sub-movement analysis shows that a minimum detectable change in smoothness as measured by *SPARC* reflects a change in delay between sub-movements that were at least 6% of the sub-movement duration or longer. Furthermore, as the metric is non-monotonic for delays less than 20% the duration of a sub-movement, it should be used with caution when studying differences in smoothness amongst fully recovered or healthy individuals. This needs to be considered when studying populations with good recovery. Finally, smoothness metrics such as *RJ* are based on rotational movements and had to be rejected as they could not be tested with the current simulations.

As QoM is studied by comparing task performance with normative values, *CM* could have been a suitable metric [70]. It is defined using correlation with a minimal jerk profile and it might be interesting to consider a *CM* measure that takes account of correlation with a velocity profile that models the reaching task. However, in our analysis, we saw that the metric might not be useful in measuring tremor or weak control of reaching movement. Additionally, the need for prior knowledge of the intended reaching task is a big drawback of the metric.

Although our simulations mimicked features of reaching in persons with stroke, such as varying duration or distance, and sub-movement segmentation [11], they cannot truly replace actual reaching by subjects who have suffered a stroke. Moreover, longitudinal studies of patterns of smoothness metrics in patients early post stroke will show how sensitive the smoothness metric over time and how these values relate to values measured in healthy age- and gender-matched subjects. We performed this analysis in our companion paper [71], where SPARC was seen to be responsive to change over time in the early phase post stroke and longitudinally associated with clinical measures of motor impairment within subjects.

## Conclusion

We recommend the use of SPARC as a valid metric to measure the smoothness of the upper limb reaching after stroke. Longitudinal studies are further required to understand the relationship between the time course of recovery and smoothness early post stroke.

## Supplementary Information

**Additional file 1.** The file consists of supplementary files as follows: 1.A Search string for the systematic literature review. 1.B Modelling reach-to-grasp movement in healthy subjects. 1.C Base velocity profiles used. 1.D Mathematical definition of metrics. 1.E Metrics and cited sources. Simulations for asymmetric profile. 1.F Monotonicity analysis for the sub-movement simulation.

**Additional file 2. **This contains all images used in the Additional file 1.

**Additional file 3. **PRISMA checklist for the systematic review filled in accordance with reporting guidelines.

**Additional file 4. **This compressed folder includes all MATLAB™ scripts used in the simulation analyses, and the resulting metric values.

## Data Availability

The generated datasets and scripts supporting the conclusions of this article are included within the article and its additional file(s).

## Abbreviations

AM: Acceleration metric
ARAT: Action research arm test
CM: Correlation metric
CSM: Combined smoothness metric
DSJb: Dimensionless squared jerk [1]
DSJm: Dimensionless squared jerk [2]
DSJt: Dimensionless squared jerk [3]
FM-UE: Fugl-Meyer Motor Assessment Score for Upper Extremity
IAJ: Integrated absolute jerk
IC: Index of curvature
ICF: International classification of functioning, disability, and health
IPV: Inversed number of peaks
ISJ: Integrated squared jerk
LDAJ: Log of dimensionless absolute jerk
LDSJb: Log dimensionless squared jerk [4]
LDSJt: Log dimensionless squared jerk [5]
MAPR: Movement arrest period ratio
NOS: Number of sub-movements
NRS: Normalized reaching speed
PRISMA: Preferred Reporting Items for Systematic Reviews and Meta-Analyses
QoM: Quality of movement
RMS: Root mean squared
RMSJ: Root mean squared jerk
SM: Speed metric
SPAL: Spectral arc length 2012 [4]
SPARC: Spectral arc length [6]
SPM: Spectral method
SPMR: Spectral metric
TM: Tent metric
VAL: Velocity arc length
WMFT: Wolf motor function test

## References

[1] Balasubramanian S, Wei R, Herman R, He J. Robot-measured performance metrics in stroke rehabilitation. Proc 2009 ICME Int Conf Complex Med Eng C 2009. 2009.

[2] Marini F, Hughes CML, Squeri V, Doglio L, Moretti P, Morasso P (2017). Robotic wrist training after stroke: adaptive modulation of assistance in pediatric rehabilitation. Rob Auton Syst.

[3] Teulings HL, Contreras-Vidal JL, Stelmach GE, Adler CH (1997). Parkinsonism reduces coordination of fingers, wrist, and arm in fine motor control. Exp Neurol.

[4] Balasubramanian S, Melendez-Calderon A, Burdet E (2012). A robust and sensitive metric for quantifying movement smoothness. IEEE Trans Biomed Eng.

[5] van Kordelaar J, van Wegen EEH, Kwakkel G (2014). Impact of time on quality of motor control of the paretic upper limb after stroke. Arch Phys Med Rehabil.

[6] Balasubramanian S, Melendez-Calderon A, Roby-Brami A, Burdet E (2015). On the analysis of movement smoothness. J Neuroeng Rehabil.

[7] Langhorne P, Bernhardt J, Kwakkel G (2011). Stroke rehabilitation. Lancet Elsevier.

[8] Sacco RL, Kasner SE, Broderick JP, Caplan LR, Connors JJ, Culebras A (2013). An updated definition of stroke for the 21st century: a statement for healthcare professionals from the American heart association/American stroke association. Stroke.

[9] Feigin VL, Forouzanfar MH, Krishnamurthi R, Mensah GA, Connor M, Bennett DA (2014). Global and regional burden of stroke during 1990–2010: findings from the Global Burden of Disease Study 2010. Lancet.

[10] Twitchell TE (1951). The restoration of motor function following hemiplegia in man. Brain.

[11] Cirstea MC, Levin MF (2000). Compensatory strategies for reaching in stroke. Brain.

[12] Bernhardt J, Borschmann KN, Kwakkel G, Burridge JH, Eng JJ, Walker MF (2019). Setting the scene for the second stroke recovery and rehabilitation roundtable. Int J Stroke.

[13] Schwarz A, Kanzler CM, Lambercy O, Luft AR, Veerbeek JM (2019). Systematic review on kinematic assessments of upper limb movements after stroke. Stroke.

[14] Rohrer B, Fasoli S, Krebs HI, Hughes R, Volpe B, Frontera WR (2002). Movement smoothness changes during stroke recovery. J Neurosci Soc Neurosci.

[15] Reinkensmeyer DJ, Burdet E, Casadio M, Krakauer JW, Kwakkel G, Lang CE (2016). Computational neurorehabilitation: modeling plasticity and learning to predict recovery. J Neuroeng Rehabil.

[16] Hogan N, Sternad D (2009). Sensitivity of smoothness measures to movement duration, amplitude, and arrests. J Mot Behav.

[17] Kiely J, Pickering C, Collins DJ. Smoothness: an unexplored window into coordinated running proficiency. Sport Med Open. 2019;5. 10.1186/s40798-019-0215-y PMC6842378 31707492

[18] Schwartz AB (2016). Leading edge perspective movement: how the brain communicates with the world. Cell.

[19] Shumway-Cook A, Woollacott MH. Motor control: translating research into clinical practice. Lippincott Williams & Wilkins; 2007.

[20] Krylow AM, Zev RW (1997). Role of intrinsic muscle properties in producing smooth movements. IEEE Trans Biomed Eng.

[21] Talelli P, Greenwood RJ, Rothwell JC (2006). Arm function after stroke: neurophysiological correlates and recovery mechanisms assessed by transcranial magnetic stimulation. Clin Neurophysiol.

[22] Melendez-Calderon A, Shirota C, Balasubramanian S (2021). Estimating movement smoothness from inertial measurement units. Front Bioeng Biotechnol.

[23] Feinstein AH, Cannon HM (2001). Fidelity, verifiability, and validity of simulation: constructs for evaluation. Dev Bus Simul Exp Learn.

[24] World Health Organization. Towards a common language for functioning, disability and health: ICF. Int Classif. 2002.

[25] Moher D, Liberati A, Tetzlaff J, Altman DG (2009). Preferred reporting items for systematic reviews and meta-analyses: the PRISMA statement. J Clin Epidemiol.

[26] WHO. ICF Classifications [Internet]. 2017 [cited 2020 Nov 4]. https://apps.who.int/classifications/icfbrowser/.

[27] Flash T, Hogan N (1985). The coordination of arm movements: an experimentally confirmed mathematical model. J Neurosci.

[28] Hughes CML, Mäueler B, Tepper H, Seegelke C (2013). Interlimb coordination during a cooperative bimanual object manipulation task. Laterality.

[29] Elias GJB, Namasivayam AA, Lozano AM (2018). Deep brain stimulation for stroke: current uses and future directions. Brain Stimul Elsevier Ltd.

[30] Lang CE, Wagner JM, Edwards DF, Sahrmann SA, Dromerick AW (2006). Recovery of grasp versus reach in people with hemiparesis poststroke. Neurorehabil Neural Repair.

[31] Rohrer B, Fasoli S, Krebs HI, Volpe B, Frontera WR, Stein J (2004). Submovements grow larger, fewer, and more blended during stroke recovery. Mot Control.

[32] Ouzzani M, Hammady H, Fedorowicz Z, Elmagarmid A (2016). Rayyan-a web and mobile app for systematic reviews. Syst Rev.

[33] Bigoni M, Baudo S, Cimolin V, Cau N, Galli M, Pianta L (2016). Does kinematics add meaningful information to clinical assessment in post-stroke upper limb rehabilitation? A case report. J Phys Ther Sci.

[34] Beppu H, Suda M, Tanaka R (1984). Analysis of cerebellar motor disorders by visually guided elbow tracking movement. Brain.

[35] Mazzoleni S, Filippi M, Carrozza MC, Posteraro F, Puzzolante L, Falchi E. Robot-aided therapy on the upper limb of subacute and chronic stroke patients: a biomechanical approach. Proc 2011 IEEE Int Conf Rehabil Robot. 2011. p. 1–6. 10.1109/ICORR.2011.5975422 22275623

[36] Rohrer B, Hogan N (2006). Avoiding spurious submovement decompositions II: a scattershot algorithm. Biol Cybern.

[37] Liebermann DG, Levin MF, McIntyre J, Weiss PL, Berman S. Arm path fragmentation and spatiotemporal features of hand reaching in healthy subjects and stroke patients. Proc 2010 Annu Int Conf IEEE Eng Med Biol. IEEE; 2010. p. 5242–5. 10.1109/IEMBS.2010.5626297 21096047

[38] Krebs HI, Volpe BT, Palazzo J, Rohrer B, Ferraro M, Fasoli S, et al. Robot aided neuro-rehabilitation in stroke: Interim results on follow-up of 76 patients and on movement indices. Integr Assist Technol Inf Age. IOS Press; 2001. p. 45–59.

[39] Brooks VB (1974). Introductory lecture to session III: some examples of programmed limb movements. Brain Res.

[40] Kahn LE, Zygman ML, Rymer WZ, Reinkensmeyer DJ. Robot-assisted reaching exercise promotes arm movement recovery in chronic hemiparetic stroke: a randomized controlled pilot study. J Neuroeng Rehabil. 2006;3. 10.1186/1743-0003-3-12 PMC1550245 16790067

[41] Abdul Rahman H, Khor KX, Yeong CF, Su ELM, Narayanan ALT (2017). The potential of iRest in measuring the hand function performance of stroke patients. Biomed Mater Eng.

[42] Bermúdez i Badia S, Cameirão MS. The Neurorehabilitation Training Toolkit (NTT): a novel worldwide accessible motor training approach for at-home rehabilitation after stroke. Stroke Res Treat. 2012;2012. 10.1155/2012/802157 PMC3350995 22619741

[43] Mohapatra S, Harrington R, Chan E, Dromerick AW, Breceda EY, Harris-Love M (2016). Role of contralesional hemisphere in paretic arm reaching in patients with severe arm paresis due to stroke: a preliminary report. Neurosci Lett.

[44] Pila O, Duret C, Laborne F, Gracies J, Bayle N, Hutin E (2017). Pattern of improvement in upper limb pointing task kinematics after a 3-month training program with robotic assistance in stroke. J Neuroeng Rehabil.

[45] Casadio M, Giannoni P, Morasso P, Sanguineti V (2009). A proof of concept study for the integration of robot therapy with physiotherapy in the treatment of stroke patients. Clin Rehabil.

[46] Hussain N, Alt Murphy M, Sunnerhagen KS (2018). Upper limb kinematics in stroke and healthy controls using target-to-target task in virtual reality. Front Neurol.

[47] Mazzoleni S, Sale P, Tiboni M, Franceschini M, Carrozza MC, Posteraro F (2013). Upper limb robot-assisted therapy in chronic and subacute stroke patients. Am J Phys Med Rehabil.

[48] Repnik E, Puh U, Goljar N, Munih M, Mihelj M (2018). Using inertial measurement units and electromyography to quantify movement during action research arm test execution. Sensors.

[49] Strohrmann C, Labruyère R, Gerber CN, van Hedel HJ, Arnrich B, Tröster G (2013). Monitoring motor capacity changes of children during rehabilitation using body-worn sensors. J NeuroEngineering Rehabil.

[50] Kostic M, Popovic M (2013). The modified drawing test for assessment of arm movement quality. J Autom Control.

[51] Rothi LJ, Horner J (1983). Restitution and substitution: two theories of recovery with application to neurobehavioral treatment. J Clin Neuropsychol.

[52] Bernhardt J, Hayward KS, Kwakkel G, Ward NS, Wolf SL, Borschmann K (2017). Agreed definitions and a shared vision for new standards in stroke recovery research: the stroke recovery and rehabilitation roundtable taskforce. Neurorehabil Neural Repair.

[53] See J, Dodakian L, Chou C, Chan V, McKenzie A, Reinkensmeyer DJ (2013). A standardized approach to the Fugl-Meyer assessment and its implications for clinical trials. Neurorehabil Neural Repair.

[54] Kwakkel G, Van Wegen E, Burridge JH, Winstein C, van Dokkum L, Alt Murphy M (2019). Standardized measurement of quality of upper limb movement after stroke: consensus-based core recommendations from the second stroke recovery and rehabilitation roundtable. Int J Stroke.

[55] Lenka A, Louis ED (2019). Revisiting the clinical phenomenology of “cerebellar tremor”: beyond the intention tremor. Cerebellum.

[56] Woodman OJ. An introduction to inertial navigation. Univ Cambridge. 2007;1–37.

[57] Mesquita IA, da Fonseca PFP, Pinheiro ARV, Velhote Correia MFP, da Silva CIC (2019). Methodological considerations for kinematic analysis of upper limbs in healthy and poststroke adults Part II: a systematic review of motion capture systems and kinematic metrics. Top Stroke Rehabil.

[58] di Biase L, Summa S, Tosi J, Taffoni F, Marano M, Rizzo AC (2018). Quantitative analysis of bradykinesia and rigidity in Parkinson’s disease. Front Neurol.

[59] Beck Y, Herman T, Brozgol M, Giladi N, Mirelman A, Hausdorff JM (2018). SPARC: a new approach to quantifying gait smoothness in patients with Parkinson’s disease. J Neuroeng Rehabil.

[60] Hreljac A (2000). Stride smoothness evaluation of runners and other athletes. Gait Posture.

[61] Choi A, Joo SB, Oh E, Mun JH (2014). Kinematic evaluation of movement smoothness in golf: relationship between the normalized jerk cost of body joints and the clubhead. Biomed Eng.

[62] Osu R, Ota K, Fujiwara T, Otaka Y, Kawato M, Liu M (2011). Quantifying the quality of hand movement in stroke patients through three-dimensional curvature. J Neuroeng Rehabil.

[63] Wininger M, NH K, Craelius W. Reformulation in the phase plane enhances smoothness rater accuracy in stroke. J Mot Behav. 2012;44:149–59. 10.1080/00222895.2012.663012 22420840

[64] Kaminski TR, Gentile AM (1989). A kinematic comparison of single and multijoint pointing movements. Exp Brain Res.

[65] Nagasaki H. Asymmetric velocity and acceleration profiles of human arm movements. Exp Brain Res. 1989;74. 10.1007/BF00248865 2924852

[66] Todorov E (2004). Optimality principle in sensorimotor control (review). Nat Neurosci.

[67] Yazdani M, Gamble G, Henderson G, Hecht-Nielsen R (2012). A simple control policy for achieving minimum jerk trajectories. Neural Netw Elsevier Ltd.

[68] Plamondon R, Alimi AM, Yergeau P, Leclerc F (1993). Modelling velocity profiles of rapid movements: a comparative study. Biol Cybern.

[69] Nelson WL (1983). Physical principles for economies of skilled movements. Biol Cybern.

[70] Kwakkel G, Lannin NA, Borschmann K, English C, Ali M, Churilov L (2017). Standardized measurement of sensorimotor recovery in stroke trials: consensus-based core recommendations from the stroke recovery and rehabilitation roundtable. Int J Stroke.

[71] Saes M, Mohamed Refai MI, van Kordelaar J, Scheltinga BL, van Beijnum B-JF, Bussmann JB (2021). Smoothness metric during reach-to-grasp after stroke. Part 2. Longitudinal association with motor impairment. J Neuro Engineering Rehabil..

[72] Simonsen D, Popovic MB, Spaich EG, Andersen OK (2017). Design and test of a Microsoft Kinect-based system for delivering adaptive visual feedback to stroke patients during training of upper limb movement. Med Biol Eng Comput.

[73] Menegoni F, Milano E, Trotti C, Galli M, Bigoni M, Baudo S (2009). Quantitative evaluation of functional limitation of upper limb movements in subjects affected by ataxia. Eur J Neurol.

[74] Duff M, Chen Y, Attygalle S, Herman J, Sundaram H, Qian G (2010). An adaptive mixed reality training system for stroke rehabilitation. IEEE Trans Neural Syst Rehabil Eng.

[75] Laczko J, Scheidt RA, Simo LS, Piovesan D (2017). Inter-joint coordination deficits revealed in the decomposition of endpoint jerk during goal-directed arm movement after stroke. IEEE Trans Neural Syst Rehabil Eng.

[76] Young RP, Marteniuk RG (1997). Acquisition of a multi-articular kicking task: jerk analysis demonstrates movements do not become smoother with learning. Hum Mov Sci.

[77] Adamovich SV, Fluet GG, Merians AS, Mathai A, Qiu Q (2009). Incorporating haptic effects into three-dimensional virtual environments to train the hemiparetic upper extremity. IEEE Trans Neural Syst Rehabil Eng.

[78] Popović MD, Kostić MD, Rodić SZ, Konstantinović LM (2014). Feedback-mediated upper extremities exercise: increasing patient motivation in poststroke rehabilitation. Biomed Res Int.

