# Supplementary material for: Smoothness metrics for reaching performance after stroke. Part 1: which one to choose?
Source: J Neuroeng Rehabil. 2021 Oct 26;18:154. doi: 10.1186/s12984-021-00949-6 (PMC8549250; doi:10.1186/s12984-021-00949-6)
Supplement: Supplementary file 1 — Additional file 1. The file consists of supplementary files as follows: 1.A Search string for the systematic literature review. 1.B Modelling reach-to-grasp movement in healthy subjects. 1.C Base velocity profiles used. 1.D Mathematical definition of metrics. 1.E Metrics and cited sources. Simulations for asymmetric profile. 1.F Monotonicity analysis for the sub-movement simulation. [file 12984_2021_949_MOESM1_ESM.docx]

Additional File 1

# AF 1.A – Search string for the systematic literature review

**Table A.1 Search string per database**

| PubMed | #1 Stroke | "Stroke"[Mesh] OR "Stroke Rehabilitation"[Mesh] OR cva[tiab] OR cvas[tiab] OR poststroke*[tiab] OR post-stroke*[tiab] OR stroke*[tiab] OR apoplex*[tiab] OR cerebrovascular diseas*[tiab] OR cerebrovascular accident*[tiab] OR cerebrovascular disorder*[tiab] OR ((brain*[tiab] OR cerebr*[tiab] OR cerebell*[tiab] OR intracran*[tiab] OR intracerebral*[tiab] OR vertebrobasilar*[tiab]) AND vascular*[tiab] AND (disease[tiab] OR diseases[tiab] OR accident*[tiab] OR disorder*[tiab])) OR ((brain*[tiab] OR cerebr*[tiab] OR cerebell*[tiab] OR intracran*[tiab] OR intracerebral*[tiab] OR vertebrobasilar*[tiab]) AND (haemorrhag*[tiab] OR hemorrhag*[tiab] OR ischemi*[tiab] OR ischaemi*[tiab] OR infarct*[tiab] OR haematoma*[tiab] OR hematoma*[tiab] OR bleed*[tiab])) |
| --- | --- | --- |
|  | #2 Kinetics and kinematics | (("Movement"[Mesh:NoExp] OR "Motion"[Mesh] OR "Spatio-Temporal Analysis"[Mesh] OR "Kinetics"[Mesh] OR Kinematic*[tiab] OR kinetic*[tiab] OR angle*[tiab] OR motion[tiab] OR acceler*[tiab] OR deceler*[tiab] OR rotation[tiab] OR velocity*[tiab] OR speed*[tiab] OR spatiotemporal[tiab])) |
|  | #3 Upper limb kinetics and kinematics | OR "Upper Extremity"[Mesh] OR Upper Extremit*[tiab] OR Upper Limb*[tiab] OR arm[tiab] OR arms[tiab] OR shoulder[tiab] OR elbow*[tiab] OR forearm*[tiab] OR wrist*[tiab] OR hand[tiab] OR hands[tiab] OR finger*[tiab] OR thumb*[tiab] |
|  | #4 Smoothness | Smooth* |
| Scopus | #1 Stroke | TITLE-ABS-KEY ( cva OR cvas OR poststroke* OR stroke* OR apoplex* OR ( ( brain* OR cerebr* OR cerebell* OR intracran* OR intracerebral* OR vertebrobasilar* ) AND vascular* AND ( disease OR diseases OR accident* OR disorder* ) ) OR ( cerebrovascular* AND ( disease OR diseases OR accident* OR disorder* ) ) OR ( ( brain* OR cerebr* OR cerebell* OR intracran* OR intracerebral* OR vertebrobasilar* ) AND ( haemorrhag* OR hemorrhag* OR ischemi* OR ischaemi* OR infarct* OR haematoma* OR hematoma* OR bleed* ) ) ) |
|  | #2 Kinetics and kinematics | TITLE-ABS-KEY ( movement OR motion OR kinematic* OR kinetic* OR angle* OR motion OR acceler* OR deceler* OR rotation OR velocity* OR speed* OR spatiotemporal ) |
|  | #3 Upper limb kinetics and kinematics | TITLE-ABS-KEY ( "Upper Extremit*" OR "Upper Limb*" OR arm OR arms OR shoulder OR elbow* OR forearm* OR wrist* OR hand OR hands OR finger* OR thumb* ) |
|  | #4 Smoothness | TITLE-ABS-KEY ( smooth* ) |
| Cochrane | #1 Stroke | cva OR cvas OR poststroke* OR stroke* OR apoplex* OR ((brain* OR cerebr* OR cerebell* OR intracran* OR intracerebral* OR vertebrobasilar*) AND vascular* AND (disease OR diseases OR accident* OR disorder*)) OR (cerebrovascular* AND (disease OR diseases OR accident* OR disorder*)) OR ((brain* OR cerebr* OR cerebell* OR intracran* OR intracerebral* OR vertebrobasilar*) AND (haemorrhag* OR hemorrhag* OR ischemi* OR ischaemi* OR infarct* OR haematoma* OR hematoma* OR bleed*)) |
|  | #2 Kinetics and kinematics | reach* OR coordination OR grasp* OR grip* OR "Upper Extremit*" OR "Upper Limb*" OR arm OR arms OR shoulder OR elbow* OR forearm* OR wrist* OR hand OR hands OR finger* OR thumb* |
|  | #3 Upper limb kinetics and kinematics | Movement OR Motion OR Mechanical OR biomechanic* OR Kinematic* OR kinetic* OR angle* OR motion OR acceler* OR deceler* OR rotation OR velocity* OR speed* OR spatiotemporal |
|  | #4 Smoothness | Smooth* |
| Embase | #1 Stroke | 'cerebrovascular accident'/exp OR cva:ab,ti OR cvas:ab,ti OR stroke:ab,ti OR apoplex*:ab,ti OR poststroke*:ab,ti OR ((brain*:ab,ti OR cerebr*:ab,ti OR cerebell*:ab,ti OR intracran*:ab,ti OR intracerebral*:ab,ti OR vertebrobasilar*:ab,ti) AND vascular*:ab,ti AND (disease:ab,ti OR diseases:ab,ti OR accident*:ab,ti OR disorder*:ab,ti)) OR (cerebrovascular*:ab,ti AND (disease:ab,ti OR diseases:ab,ti OR accident*:ab,ti OR disorder*:ab,ti)) OR ((brain*:ab,ti OR cerebr*:ab,ti OR cerebell*:ab,ti OR intracran*:ab,ti OR intracerebral*:ab,ti OR vertebrobasilar*:ab,ti) AND (haemorrhag*:ab,ti OR hemorrhag*:ab,ti OR ischemi*:ab,ti OR ischaemi*:ab,ti OR infarct*:ab,ti OR haematoma*:ab,ti OR hematoma*:ab,ti OR bleed*:ab,ti)) |
|  | #2 Kinetics and kinematics | reach*:ti,ab OR coordination:ti,ab OR grasp*:ti,ab OR grip*:ti,ab OR 'upper limb'/exp OR 'Upper Extremit*':ti,ab OR 'Upper Limb*':ti,ab OR arm:ti,ab OR arms:ti,ab OR shoulder:ti,ab OR elbow*:ti,ab OR forearm*:ti,ab OR wrist*:ti,ab OR hand:ti,ab OR hands:ti,ab OR finger*:ti,ab OR thumb*:ti,ab |
|  | #3 Upper limb kinetics and kinematics | 'movement (physiology)'/de OR 'limb movement'/de OR 'arm movement'/exp OR 'hand movement'/exp OR 'motion'/de OR 'velocity'/exp OR 'mechanics'/de OR 'biomechanics'/exp OR 'force'/exp OR 'kinematics'/exp OR 'kinetics'/de OR 'torque'/exp OR 'temporal analysis'/exp OR 'spatial analysis'/de OR torque*:ti,ab OR biomechanic*:ti,ab OR Kinematic*:ti,ab OR kinetic*:ti,ab OR angle*:ti,ab OR force*:ti,ab OR motion:ti,ab OR acceler*:ti,ab OR deceler*:ti,ab OR rotation:ti,ab OR velocity*:ti,ab OR speed*:ti,ab OR spatiotemporal:ti,ab |
|  | #4 Smoothness | Smooth*:ti,ab |
| CINAHL | #1 Stroke | (cva OR cvas OR poststroke* OR stroke* OR apoplex) OR ( brain* OR cerebr* OR cerebell* OR intracran* OR intracerebral* OR vertebrobasilar* ) AND vascular* AND ( disease OR diseases OR accident* OR disorder* ) OR (cerebrovascular* AND ( disease OR diseases OR accident* OR disorder* ) ) OR ( brain* OR cerebr* OR cerebell* OR intracran* OR intracerebral* OR vertebrobasilar* ) AND ( haemorrhag* OR hemorrhag* OR ischemi* OR ischaemi* OR infarct* OR haematoma* OR hematoma* OR bleed* ) |
|  | #2 Kinetics and kinematics | reach* OR coordination OR grasp* OR grip* OR "Upper Extremit*" OR "Upper Limb*" OR arm OR arms OR shoulder OR elbow* OR forearm* OR wrist* OR hand OR hands OR finger* OR thumb* |
|  | #3 Upper limb kinetics and kinematics | Movement OR Motion OR Mechanical OR biomechanic* OR Kinematic* OR kinetic* OR angle* OR motion OR acceler* OR deceler* OR rotation OR velocity* OR speed* OR spatiotemporal |
|  | #4 Smoothness | smooth* |

All articles with #1 AND #2 AND #3 AND #4 NOT ('animal'/exp NOT 'human'/exp) were retrieved.

# AF 1.B – Modelling reach-to-grasp movement in healthy subjects

A minimal jerk model correlates well with healthy subjects in 2D unconstrained point-to-point reaching movements [1]. Reach-to-grasp movement is commonly used to assess movement quality as part of clinical tests such as ARAT and FM-UE. However, reach-to-grasp does not follow a minimal jerk profile [2]. Therefore, here, we model a base velocity profile for reach-to-grasp movements required for testing the different simulations proposed in the paper. For this, data from the EXPLICIT study containing 12 healthy subjects performing reach-to-grasp a block as part of the ARAT using the dominant hand was used. The study was registered at the Netherlands National Trial Register (NTR1424), approved by the Medical Ethics Committee of the VU University Medical Centre, Amsterdam, The Netherlands and carried out in accordance with the Code of Ethics of the World Medical Association, Declaration of Helsinki. The average age of the subjects was 64.2 $\pm$ 5.8 years, and seven of them identified as males. Fig B.1 shows all 28 trials of reach-to-grasp movement performed by a healthy subject. The profile, unlike a minimal jerk model is skewed and the peak speed is not at half of the reaching duration.

**Fig. B.1:** Trials of the dominant hand of a single healthy subject performing reaching tasks.

First, we attempted to model a reaching profile by averaging reaching data from all healthy subjects. However, as resting hand velocity among subjects was not exactly zero, there were minor differences in the start and end velocities among subjects. Therefore, the average profile did not have a zero velocity and zero acceleration at the end and beginning of the movement. Therefore, using Fig. B.1, we defined requirements for the shape of the asymmetric profile, and fitted a curve through these specific points. For instance, the velocity at the beginning and end of the movement should be zero. Further, the peak value of velocity should be at 32.7% of the total reaching time. Therefore, the following coordinates are available: (0; 0), (0.327; 1) and (1; 0). As three coordinates are known, it is possible to fit these points using a polynomial. With this procedure, the derivatives can also be taken in to account. The following six conditions were created for the coordinates and derivatives. MATLAB^TM^ 2015b was used for the analysis.

**Coordinates (x; y) Derivatives (x; dy/dx)**

1. (0; 0) **(2)** (0; 0)
2. (0.327; 1) **(4)** (0.327; 0)
3. (1; 0) **(6)** (1; 0)

As there are six conditions, a polynomial with six variables is expected to meet these conditions. This polynomial would be in the form of $y\left( x \right)=a+bx+cx^{2}+dx^{3}+ ex^{4}+ fx^{5}$.

Using the conditions stated above, the following equations are generated:

1. $a=0$
2. $b=0$
3. $a+b0.327+c{0.327}^{2}+d{0.327}^{3}+ e{0.327}^{4}+ f{0.327}^{5}=1$
4. $b+2c0.327+3d{0.327}^{2}+ 4e{0.327}^{3}+ 5f{0.327}^{4}=0$
5. $a+b+c+d+ e+ f= 0$
6. $b+2c+3d+ 4e+ 5f= 0$

Solving the set of equations, a solution was found, as can be seen in Fig. B.2 (*g* = 0). Although this velocity profile satisfies all the requirements, it does not have the desired profile of Fig. B.1. The additional variable *g* was tuned to improve the profile. The new polynomial equation is therefore $y\left( x \right)=a+bx+cx^{2}+dx^{3}+ ex^{4}+ fx^{5}+gx^{6}$.

The equations stated above are now solved for this new equation. However, multiple solutions were found as there is redundancy in this set of solutions (seven variables, six equations). To optimize the value for parameter *g*, an error cost function is calculated and optimized

$error= \frac{\int\left| v-v_{fit} \right|dt}{\int v dt}$, with *v*, the average velocity profile of subject 1 and *v_fit_* the modelled polynomial velocity profile. *v_fit_* was adjusted based on the distance and duration of the reaching movement *v*. *v* is derived by normalizing all trials to movement distance and duration. The error is minimized using an interior-point algorithm (implemented using MATLAB^TM^ function *fmincon*). A *g* value of 54 was found to be most optimum. This fit will be referred to as ‘*Polynomial 1*’.


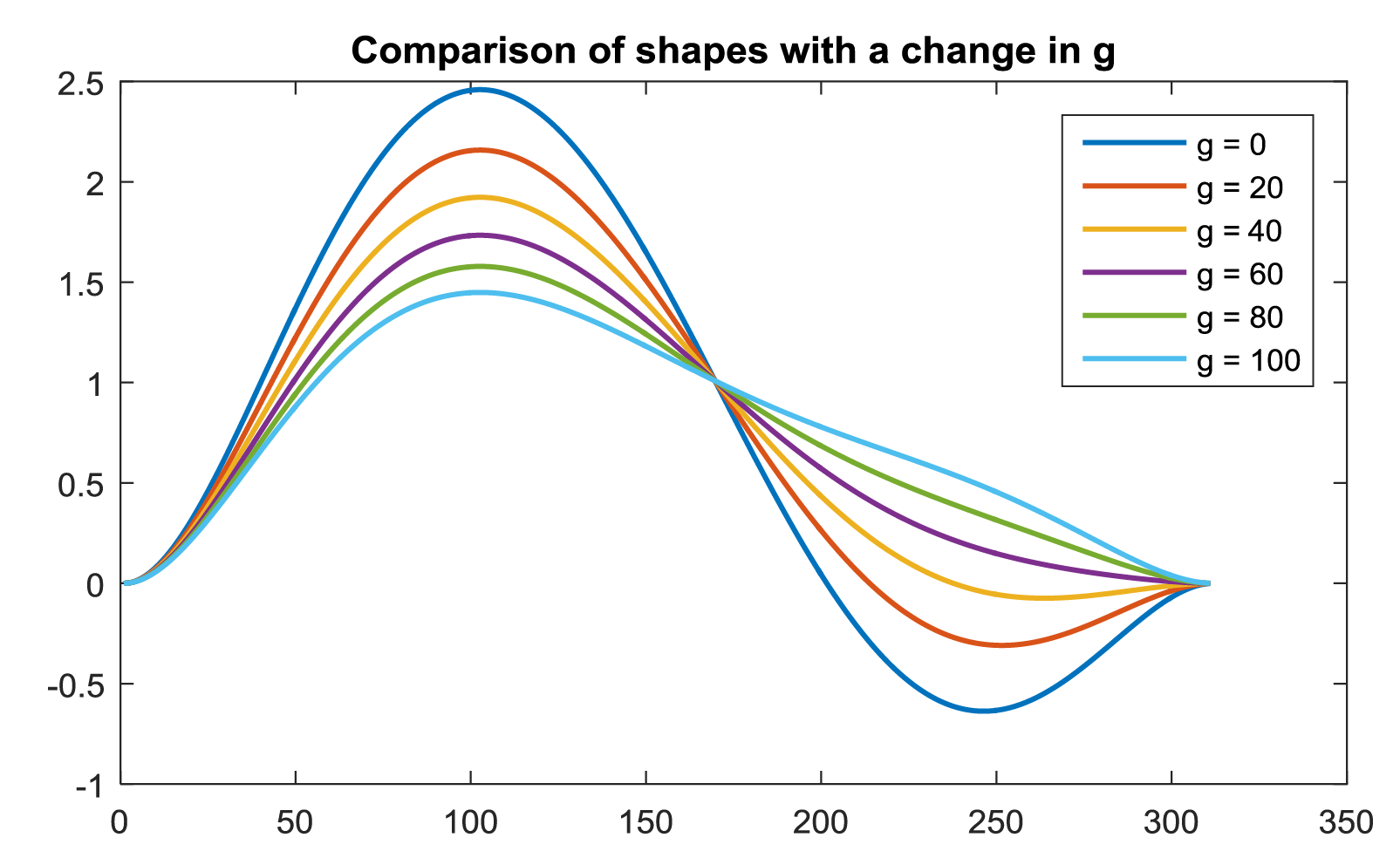


**Fig. B.2:** Influence of varying the parameter ‘g’ on the polynomial fit.

To generalize the polynomial model for all subjects, all trials of all healthy subjects were normalized, and averaged after removing outlies. Using the average profile, the error cost function was minimized by tuning variable *g*, and also the location of the peak value, using *fmincon.* This resulted in a value of 81.5 for the *g* parameter and 0.3157 for the peak location. The error between the new found profile and the normalized mean velocity profile was 0.04. This model had a lower steep slope following the peak velocity, and this polynomial will be referred as ‘*Polynomial 2*’.

It was found that the difference between the two polynomial profiles is not that large. Over all trials, *Polynomial 1*, had an average error of 0.2369 while *Polynomial 2* had an slightly lower average error of 0.2169. *Polynomial 2* will be used in the analysis and can also been seen in Fig. C.1.

# AF 1.C – Base velocity profiles used


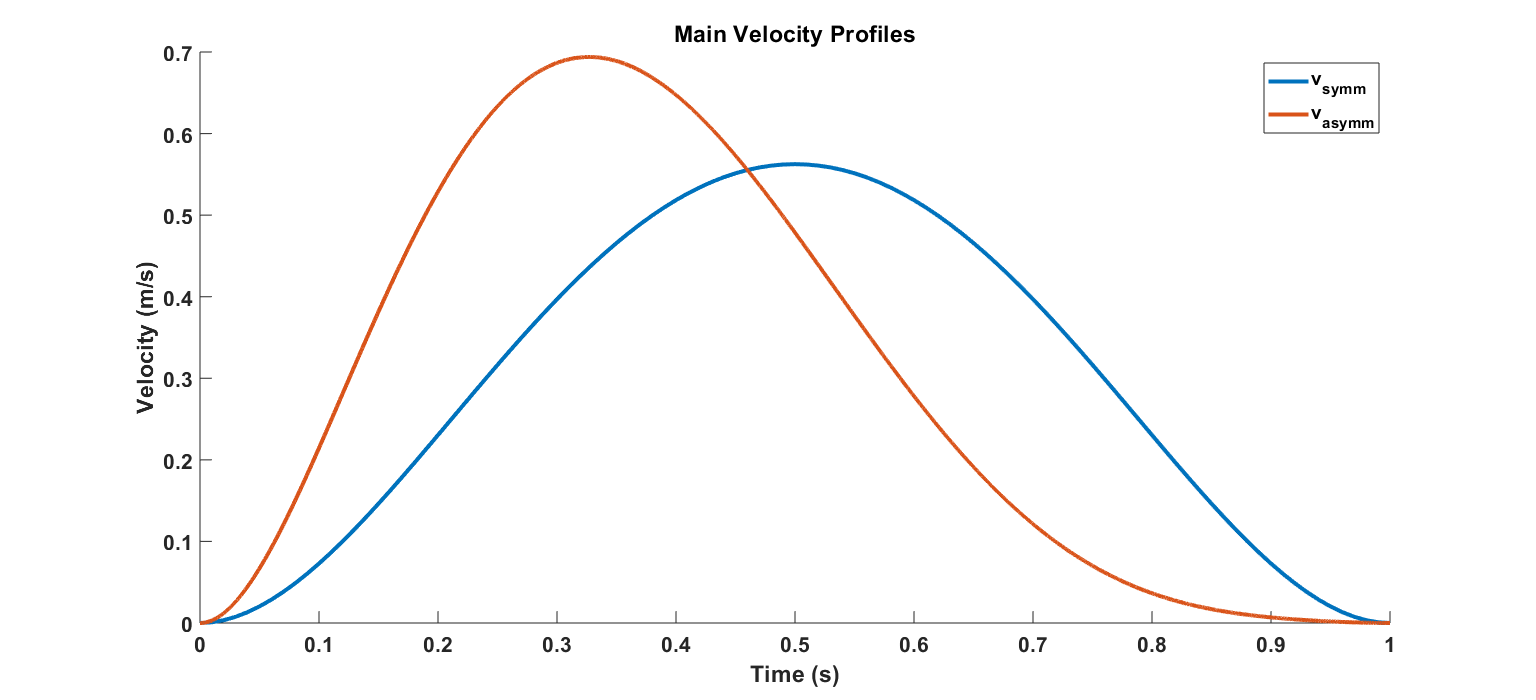


**Fig. C.1:** The base velocity profiles (symmetric and asymmetric) with a reaching duration of 1 s and distance 0.3 m are shown.

# AF 1.D – Mathematical definition of metrics

- *Number of sub-movements (NOS)* [3]: This algorithm fits the velocity profile by a combination of minimal jerk velocity profiles. This is done by using an interior-point algorithm (MATLAB’s function ***fmincon***) and minimizing the error function:


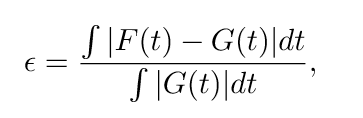
 where G(t) is the movement speed profile and F(t) is the fitted speed profile.


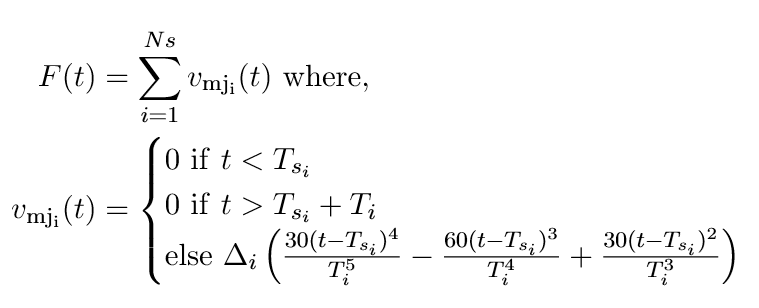


With Ns the number of sub-movements, v_mj_ the minimal jerk speed profile, ∆ the sub-movement distance, T the sub-movement duration and T_s_ the sub-movement time shift. Subscript i denotes the i^th^ sub-movement. ∆, T_s_ and T are minimized, while the function was ten times initialized at random points within the solution space, with Ns= 1. For ∆ the solution space is between 0 and 1 m, for T between 0.01 and 3 s and for T_s_ between 0 s and the total duration of the movement. The sampling frequency used was 100 Hz.

The optimization ended if the error was less than 0.02. In other cases, the number of sub movements is increased, and the algorithm is iterated once more. In case more than 7 sub movements were required, the algorithm was aborted. In these extreme situations (presence of added noise, or sinusoidal disturbances), the algorithm fails to converge to the right solution. Finally, *NOS* gives the number of subtracted sub-movements Ns, which is the measure for smoothness.

We analyzed the optimization algorithm and found that the time taken for the optimization increased with the number of sub-movements. As the number of submovements increased, the number of iterations required for optimization also increased. The computational costs were also high. For instance, when a movement was made up of 8 sub-movements, an optimum solution was found only once out of 250 iterations of the optimization. This took 21 minutes. Although further constraints can be explored for the algorithm, the choices made for selecting the boundary criteria influences the *NOS*. These are major drawbacks to address if one wishes to improve this metric.

- *Speed metric (SM)* [4]: $SM = \frac{v_{mean}}{v_{peak}}$, where v_mean_ and v_peak_ are respectively the mean and peak velocity of the whole movement.
- *Normalized reaching speed (NRS)* [5]: $NRS=\frac{v_{peak}-v_{mean}}{v_{peak}}$
- *Movement arrest period ratio* (*MAPR*) [6]: $MAPR = \frac{t\left( v\geq F\cdot v_{peak} \right)}{T},$ where F is the fraction that is taken from the peak velocity to calculate the threshold and T is the total duration. Rohrer and colleagues [4] used a F-value of 0.1.
-
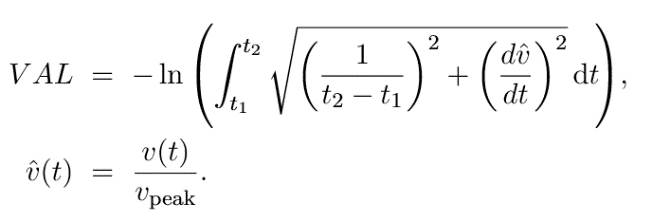
*Velocity arc length (VAL)* [7]:

where *t*_1_ and *t*_2_ are the time points at the start and end of the movement.

- *Correlation metric (CM)*[8]: First, the minimum jerk speed profile is calculated by

$v_{\text{mj}}\left( t \right)=\Delta\left( \frac{30t^{4}}{T^{5}}-\frac{60t^{3}}{T^{4}}+\frac{30t^{2}}{T^{3}} \right),$ $v_{norm}\left( t \right)=\frac{v\left( t \right)}{v_{peak}},$

where *v*(*t*) is the hand speed, *v*_mj_(*t*) is the minimal jerk speed profile. ∆ is the distance of the reaching movement. T is the duration of the reaching movement. Then, the correlation coefficient is calculated in the standard form as

$$\rho=\frac{\sum\left[ \left( v_{norm}- \bar{v}_{norm} \right)\left( v_{mj}-\bar{v}_{mj} \right) \right]}{\sqrt{\left( \sum\left[ \left( v_{norm}-\bar{v}_{norm} \right)^{2}\sum\left( v_{mj}-\bar{v}_{mj} \right)^{2} \right] \right)}},$$

Where $\bar{v}_{norm}$and $\bar{v}_{mj}$are the mean values of the normalized hand speed and minimum jerk speed profile.

As the CM ranges between [-1.0 1.0], a value between [0.3 1] shows moderate to strong correlation with a minimum jerk profile. However, any values between [-1.0 - 0.3] might suggest that reaching profile has a moderate to strong correlation in the negative direction. This could most likely occur if the measurement system is not properly calibrated or if the participant is performing a reverse reach movement (which is unlikely). Any value between [-0.3 0.3] shows that the movement performed is very noisy or is quite intermittent and thereby of very low smoothness. It might be of interest to consider a CM measure that takes account of correlation with a velocity profile that models the reaching task, for instance the asymmetric profile for reach-to-grasp movements.

- *Peaks metric (Peaks)* [9]: This metric counts the number of *v*_maxima_*,* where v_maxima_ is defined as $v\left( t \right): \dot{v}(t)=0 \text{and }\ddot{v}(t)<0,$ where $v\left( t \right),\dot{v}(t)$, and $\ddot{v}(t)$ are respectively the first, second and third time derivative of position.
- *Number of Peaks normalized by movement duration (NPt)* [10]*:* ${PM}_{md}= \frac{PM}{T}$
- *Number of Peaks normalized by movement distance (NPd)* [11]*:* ${PM}_{ps}=\frac{PM}{\Delta}$.
- *Inverse number of peaks and valleys (IPV)* [12] is defined by

${PM}_{inv}= \frac{1}{\left( PM*2 \right)-1},$ where PM is the number of peaks, defined earlier, v_peak_ is the peak velocity within the movement and T is the total movement duration. Note here that the number of peaks and valleys is defined as $\left( PM*2 \right)-1$.

- *Acceleration metric (AM)* [5]: $AM=\frac{\ddot{x}_{mean}}{\ddot{x}_{peak}},$ where $\ddot{x}$ is the second derivative of *x*(*t*) with respect to time, which is the acceleration.
- *Integrated absolute jerk (IAJ)* [13]: $\eta_{\text{iaj}}=\int_{t1}^{t2} \left| x\left( t \right) \right|dt$, where $x\left( t \right)$ is the third derivative of *x*(*t*) with respect to time, which is the jerk.
- *Mean absolute jerk (MAJ)* [14]: $\eta_{\text{maj}}=\frac{1}{t_{2}-t_{1}}\int_{t1}^{t2} \left| x\left( t \right) \right|dt$.
- *Mean absolute jerk, normalized by peak speed (MAJPS)* [4]:

$$\eta_{\text{majps}}=\frac{1}{v_{peak}\left( t_{2}-t_{1} \right)}\int_{t_{1}}^{t_{2}} x\left( t \right)^{2}dt.$$

- *Integrated squared jerk (ISJ)* [15]: $\eta_{\text{isj}}=\int_{t_{1}}^{t_{2}} x\left( t \right)^{2}dt.$
- *Root mean squared jerk (RMSJ)* [16]: $\eta_{\text{rmsj}}=\sqrt{\frac{1}{\left( t_{2}-t_{1} \right)}\int_{t_{1}}^{t_{2}} x\left( t \right)^{2}dt.}$
- *Normalized integrated jerk (NIJ)* [17]:

$$\eta_{\text{nij}} =\sqrt{\frac{1}{2}\frac{\left( t_{2}-t_{1} \right)^{3}}{v_{\text{mean}}^{2}}}\int_{t_{1}}^{t_{2}} x\left( t \right)^{2}dt.$$

- *Dimensionless squared jerk (DSJt)* [18]:

$$\eta_{\text{dsj-t}} =\sqrt{\frac{1}{2}\frac{\left( t_{2}-t_{1} \right)^{3}}{v_{\text{mean}}^{2}}\int_{t_{1}}^{t_{2}} x\left( t \right)^{2}dt.}$$

- *Log dimensionless squared jerk (LDSJt)* [19]:

$$\eta_{\text{ldsj-t}} =\ln\left( \sqrt{\frac{1}{2}\frac{\left( t_{2}-t_{1} \right)^{3}}{v_{\text{mean}}^{2}}\int_{t_{1}}^{t_{2}} x\left( t \right)^{2}dt} \right).$$

- *Dimensionless squared jerk (DSJm)* [20]:

$$\eta_{\text{dsj-m}} =\frac{1}{2}\sqrt{\frac{\left( t_{2}-t_{1} \right)^{3}}{v_{\text{mean}}^{2}}\int_{t_{1}}^{t_{2}} x\left( t \right)^{2}dt,}$$

Note that this one is the same as the *DSJt* [18]. However, there is a factor 1/2 in front of the square root resulting in a value which is $\sqrt{\frac{1}{2}}$ higher.

- *Dimensionless squared jerk (DSJb)* [7]:

$$\eta_{\text{dsj-b}} =\frac{\left( t_{2}-t_{1} \right)^{3}}{v_{\text{peak}}^{2}}\int_{t_{1}}^{t_{2}} x\left( t \right)^{2}dt.$$

- *Log dimensionless squared jerk (LDSJb)* [7]*:*

$$\eta_{\text{ldsj-b}} =\ln\left( \frac{\left( t_{2}-t_{1} \right)^{3}}{v_{\text{peak}}^{2}}\int_{t_{1}}^{t_{2}} x\left( t \right)^{2}dt \right),$$

- *Rotational jerk* [21]: $\eta_{\text{rot}} =\log\sqrt{\frac{\left( t_{2}-t_{1} \right)^{5}}{2\theta^{p}}} \int_{t_{1}}^{t_{2}} \left\| \frac{d^{2}\omega(t)}{{dt}^{2}} \right\|^{2}dt,$ where t_1_ is the beginning of the movement, t_1_ the end of the movement, $\omega(t)$ is the hand angular velocity vector and parameter $\theta^{p}$ normalizes the jerk index with angular displacement of the rotation movement.
- *Spectral metric (SPMR)* [22]:

$$V\left( \omega\right)=fft\left( v\left( t \right) \right)$$

$$\bar{V}\left( \omega\right)=\frac{V\left( \omega\right)}{\sum_{\forall\omega} \{V\left( \omega\right)\}}$$

$SPMR=\begin{matrix} max \\ \forall\omega\end{matrix}\{V\left( \omega\right)\},$ Where $fft\left( v\left( t \right) \right)$ is the fast Fourier transform operation. The parameters are in this transformation are chosen such that each bin in the frequency domain is equal to 0.2Hz

- *Spectral method (SPM)* [23]:


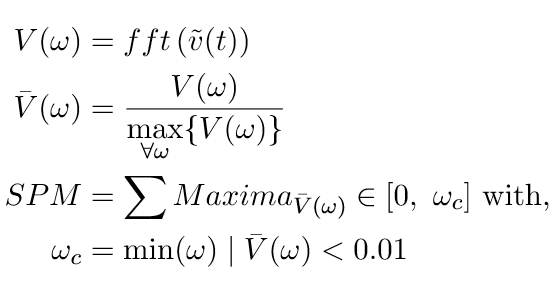


where $\tilde{v}(t)$is the zero padded version of $v(t)$, *V* (*ω*) is the Fourier magnitude spectrum of $\tilde{v}(t)$, and [0 $\omega_{c}$] is the frequency band occupied by the given movement. Before detecting the maxima, spectral smoothing was done using a moving average filter using a window size of 5 samples.

- *Spectral arc length 2012 (SPAL)* [7]*:*


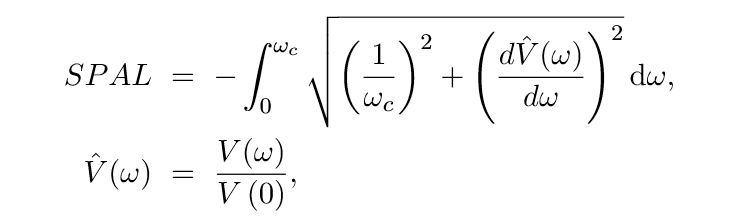


where

V

(

ω

) is the Fourier magnitude spectrum of

v

(

t

), and [0

,ω

c

] is the frequency band

occupied by the given movement

where

V

(

ω

) is the Fourier magnitude spectrum of

v

(

t

), and [0

,ω

c

] is the frequency band

occupied by the given movement

where V(ω) is the Fourier magnitude spectrum of v(t), and [0 $\omega_{c}$] is the frequency band occupied by the given movement

- *Spectral arc length (SPARC)* [24] uses the same formula as the *SPAL*. However, to determine $\omega_{c}$ the following additional formula is used


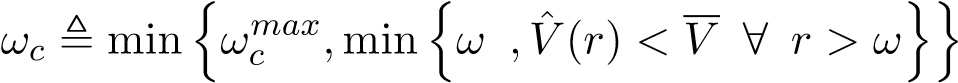
.

- *Combined smoothness metric (CSM)* [25]:

$$CSM= e^{(J_{h}+J_{i})}+e^{(P_{h}+P_{i})}+ \frac{V_{i}}{V_{h}}+\frac{T_{i}}{T_{h}} ,$$

Where $J_{i}$ is the mean negative jerk, normalized by peak velocity, $P_{i}$ is the number of peaks in the velocity profile, $V_{i}$ is the ratio of mean velocity and peak velocity and $T_{i}$ is the ratio of area under the velocity profile and its convex hull. The terms $J_{h}, P_{h}, V_{h}$ and $T_{h}$ are the normal values, determined by the authors. Respectively 1.15, 1, 0.5 and 0.9. were the values used by Kostić and Popović (2013).

# AF 1.E – Metrics and cited sources

**Table E.1 Metrics and cited sources**

| Metric | References |
| --- | --- |
| Index of curvature | [14] |
| SD_XY | [26] |
| Number of sub-movements (NOS) | [27] |
| Speed metric (SM) | [4,5,27–39] |
| Normalized reaching speed (NRS) | [5,40] |
| Movement arrest period ratio (MAPR) | [4,27,38] |
| Tent metric | [4] |
| Velocity arc length (VAL) | [24] |
| Correlation metric (CM) | [41,42] |
| Number of peaks (Peaks) | [4,5,40,43–51,7,52–61,10,62–71,12,72–81,14,82–91,29,92,31–33] |
| Number of movement units (NMU) | [90–92] |
| Number of peaks normalized by movement duration (NPt) | [10] |
| Number of peaks normalized by movement distance (NPd) | [11,89,93] |
| Inverse number of peaks and valleys (IPV) | [12] |
| Acceleration metric (AM) | [5,33] |
| Integrated absolute jerk (IAJ) | [13] |
| Mean absolute jerk (MAJ) | [14,94] |
| Mean absolute jerk normalized by peak speed (MAJPS) | [4,5,7,29,40,52,95] |
| Integrated squared jerk (ISJ) | [15] |
| Root mean squared jerk metric (RMSJ) | [96] |
| Normalized integrated jerk (NIJ) | [17] |
| Dimensionless squared jerk (DSJt) | [28,45,73,86,97–104] |
| Log dimensionless squared jerk (LDSJt) | [19] |
| Dimensionless squared jerk (DSJm) | [20] |
| Dimensionless squared jerk (DSJb) | [7] |
| Log dimensionless squared jerk (LDSJb) | [7] |
| Rotational jerk | [21] |
| Spectral metric (SPMR) | [22] |
| Spectral method (SPM) | [23] |
| Spectral arc length 2012 (SPAL) | [7,21,31,32,105–108] |
| Spectral arc length (SPARC) | [24,109] |
| Combined smoothness metric (CSM) | [110] |

# AF 1.F – Simulation Results for the Asymmetrical Profile


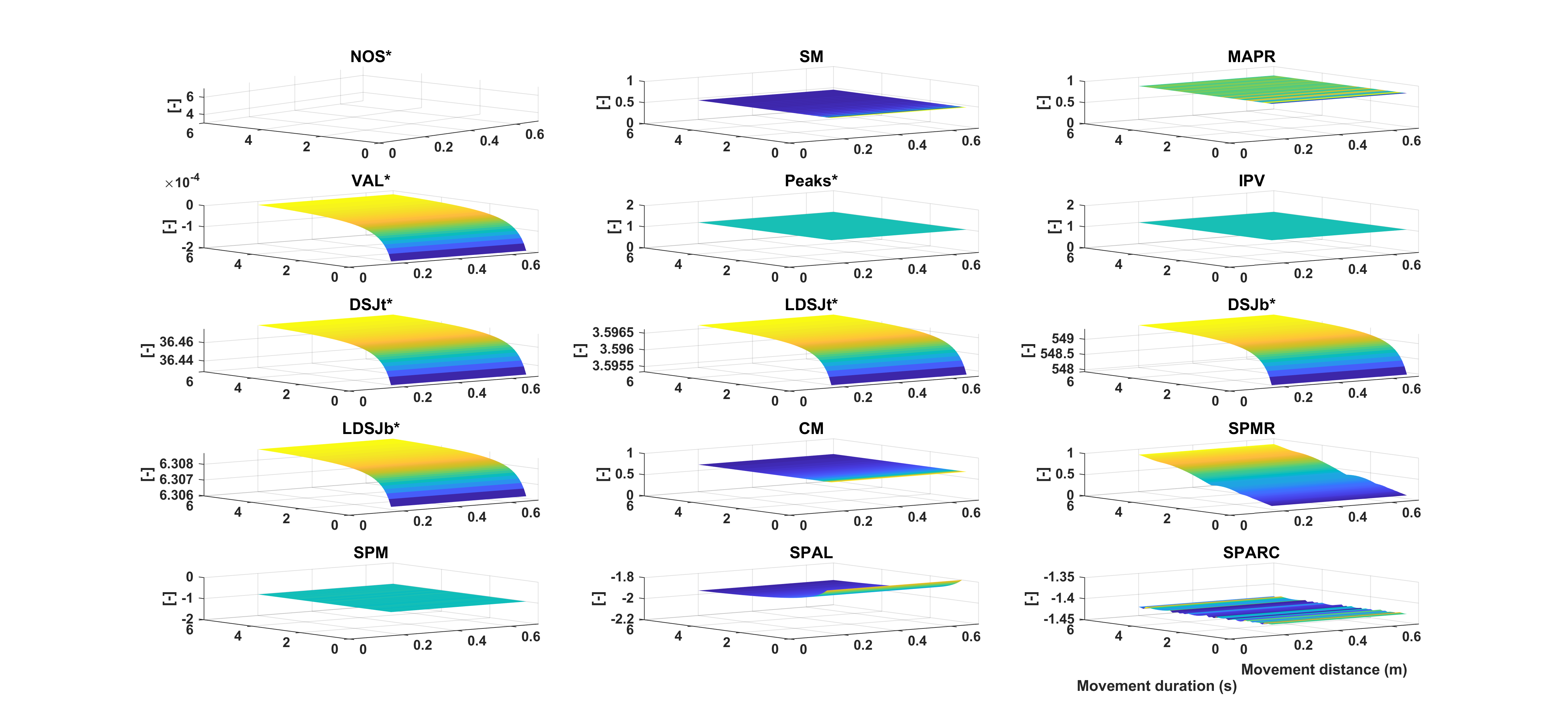


**Fig. F.1: Shape simulation.** The vertical axis represents the metric value decreasing from yellow to blue. The horizontal axes represent the movement duration and movement distance. Metrics included are *NOS** (number of sub-movements), *SM* (speed metric), *MAPR* (movement arrest period ratio), *VAL** (velocity arc length), *Peaks* (number of peaks), *IPV* (inverse of number of peaks and valleys), *DSJt** and *DSJb** (Dimensionless squared jerk), *LDSJb** and *LDSJt** (log of *DSJt** and *DSJb**), *CM* (correlation metric), *SPMR* (spectral metric), *SPM* (spectral method), *SPAL* (spectral arc length 2012), and *SPARC* (spectral arc length). By definition, the metrics with a * increase with decreasing smoothness.


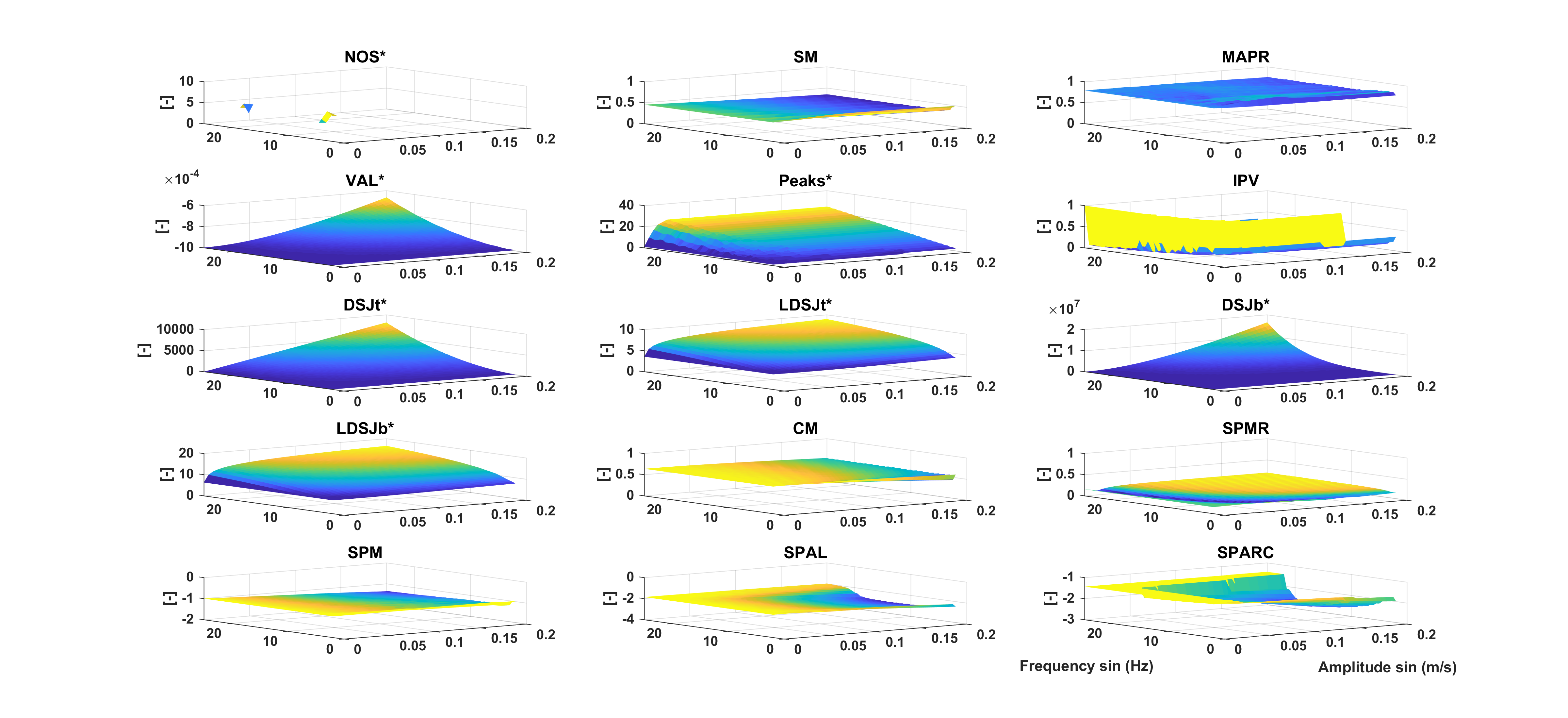


**Fig. F.2: Harmonic Disturbances.** The vertical axis represents the metric value decreasing from yellow to blue. Metrics included are *NOS** (number of sub-movements), *SM* (speed metric), *MAPR* (movement arrest period ratio), *VAL** (velocity arc length), *Peaks* (number of peaks), *IPV* (inverse of number of peaks and valleys), *DSJt** and *DSJb** (Dimensionless squared jerk), *LDSJb** and *LDSJt** (log of *DSJt** and *DSJb**), *CM* (correlation metric), *SPMR* (spectral metric), *SPM* (spectral method), *SPAL* (spectral arc length 2012), and *SPARC* (spectral arc length). By definition, the metrics with a * increase with decreasing smoothness.


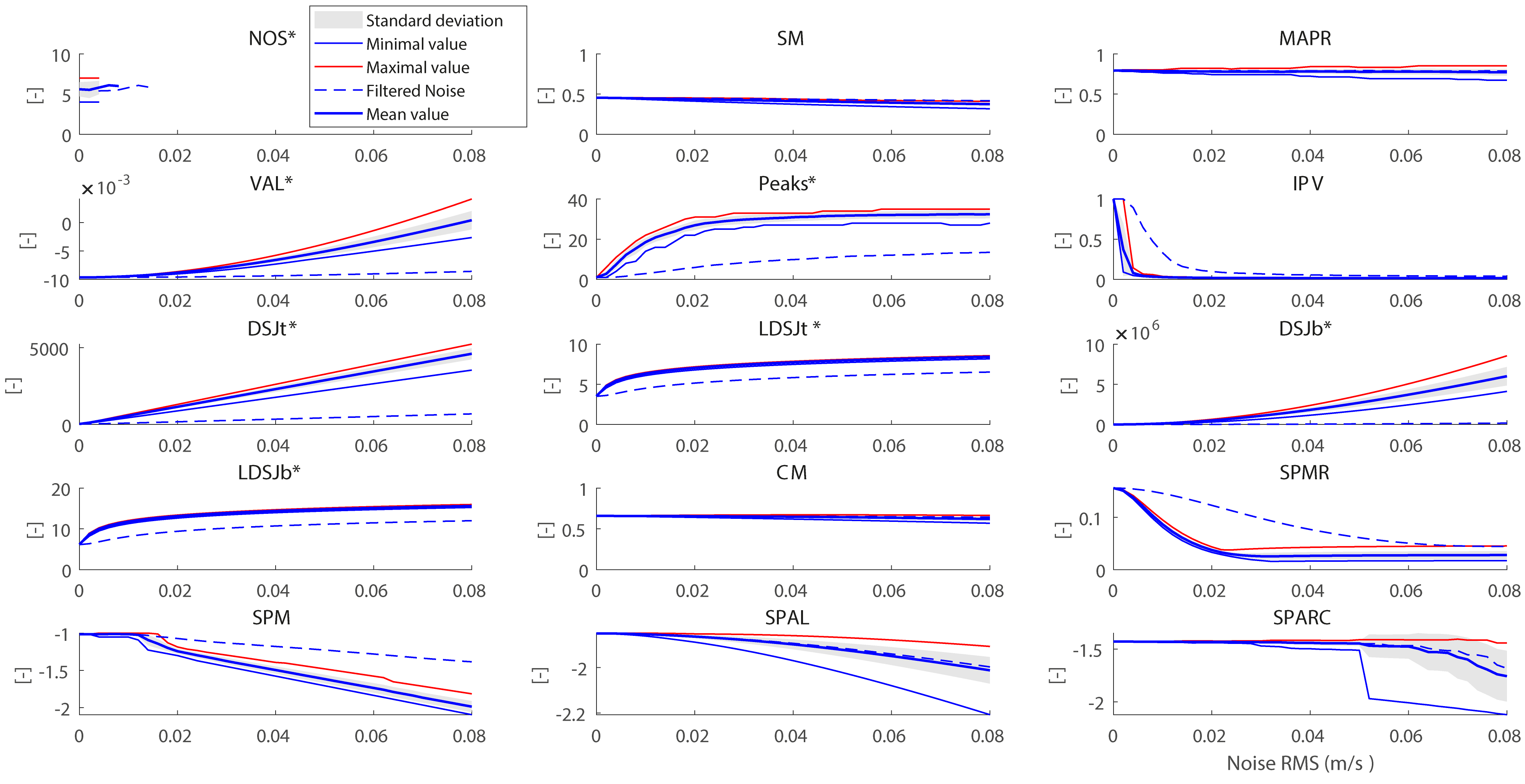


**Fig. F.3: Measurement Noise.** The thick blue line represents the mean value of 25 different realizations of the noise for each noise RMS, and shaded area is the corresponding standard deviation. The dotted lines denote the minimum and maximum value of the metric found at that RMS value. The dashed blue line shows mean value of the filtered noise sets. Metrics included are *NOS** (number of sub-movements), *SM* (speed metric), *MAPR* (movement arrest period ratio), *VAL** (velocity arc length), *Peaks* (number of peaks), *IPV* (inverse of number of peaks and valleys), *DSJt** and *DSJb** (Dimensionless squared jerk), *LDSJb** and *LDSJt** (log of *DSJt** and *DSJb**), *CM* (correlation metric), *SPMR* (spectral metric), *SPM* (spectral method), *SPAL* (spectral arc length 2012), and *SPARC* (spectral arc length). By definition, the metrics with a * increase with decreasing smoothness.


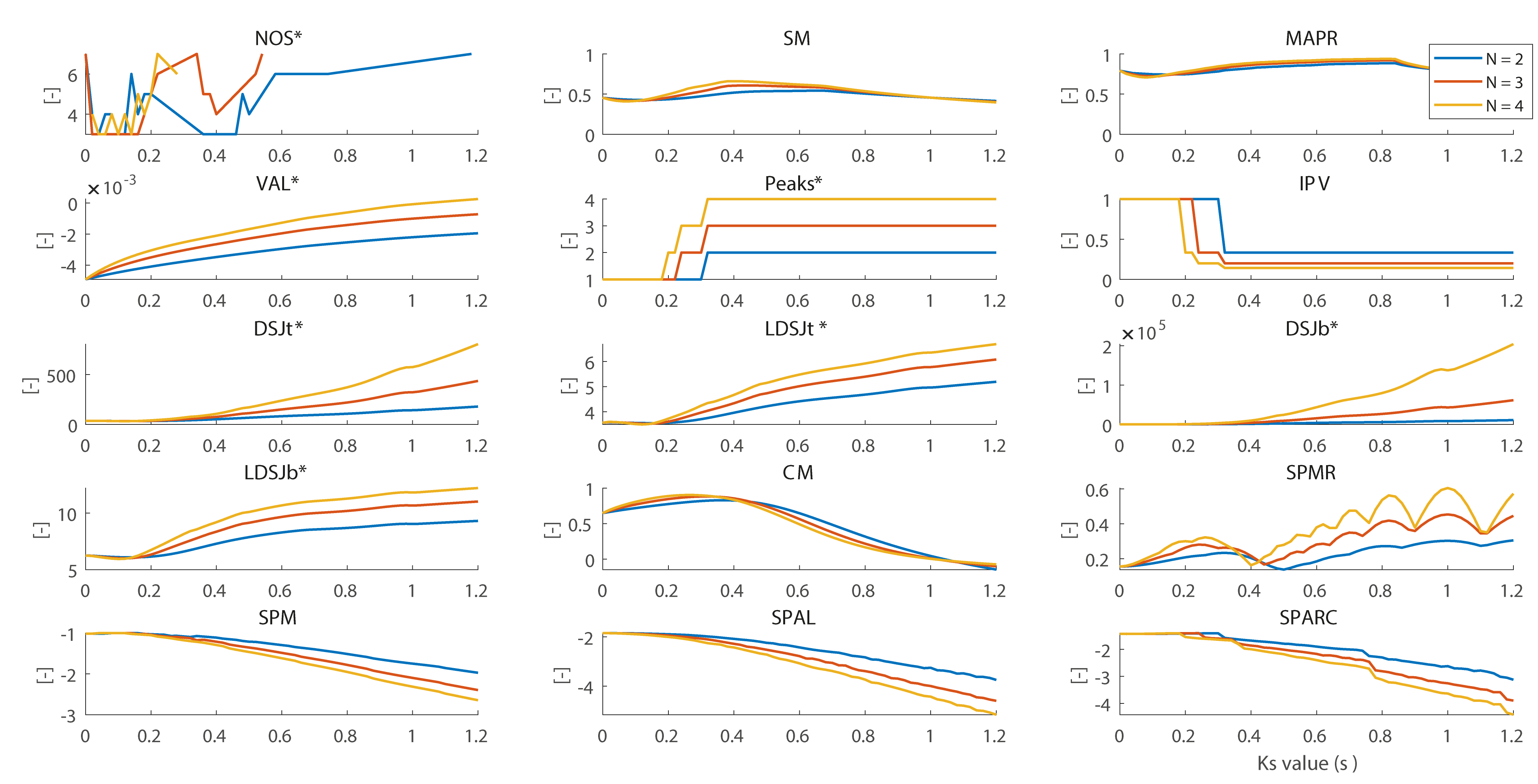


**Fig. F.4: Sub-movements simulation.** The colours denote the number of sub-movements. Metrics included are NOS* (number of sub-movements), SM (speed metric), MAPR (movement arrest period ratio), VAL* (velocity arc length), Peaks (number of peaks), IPV (inverse of number of peaks and valleys), DSJt* and DSJb* (Dimensionless squared jerk), LDSJb* and LDSJt* (log of DSJt* and DSJb*), CM (correlation metric), SPMR (spectral metric), SPM (spectral method), SPAL (spectral arc length 2012), and SPARC (spectral arc length). By definition, the metrics with a * increase with decreasing smoothness.

# AF 1.G – Monotonicity analysis for the sub-movement simulation

The monotonicity of each metric during the sub-movement simulation and the influence of varying the underlying base profiles used was assessed. In addition to the minimal jerk model, two other profiles were used for the symmetric profile: Blackman and Hann. Additionally, a lognormal distribution (mean = 0.5, standard deviation = 0.5) was designed for the asymmetric profile. All velocity profiles had a total duration of 1s, and a total reaching distance of 0.3 m. The velocity profiles are as follows:


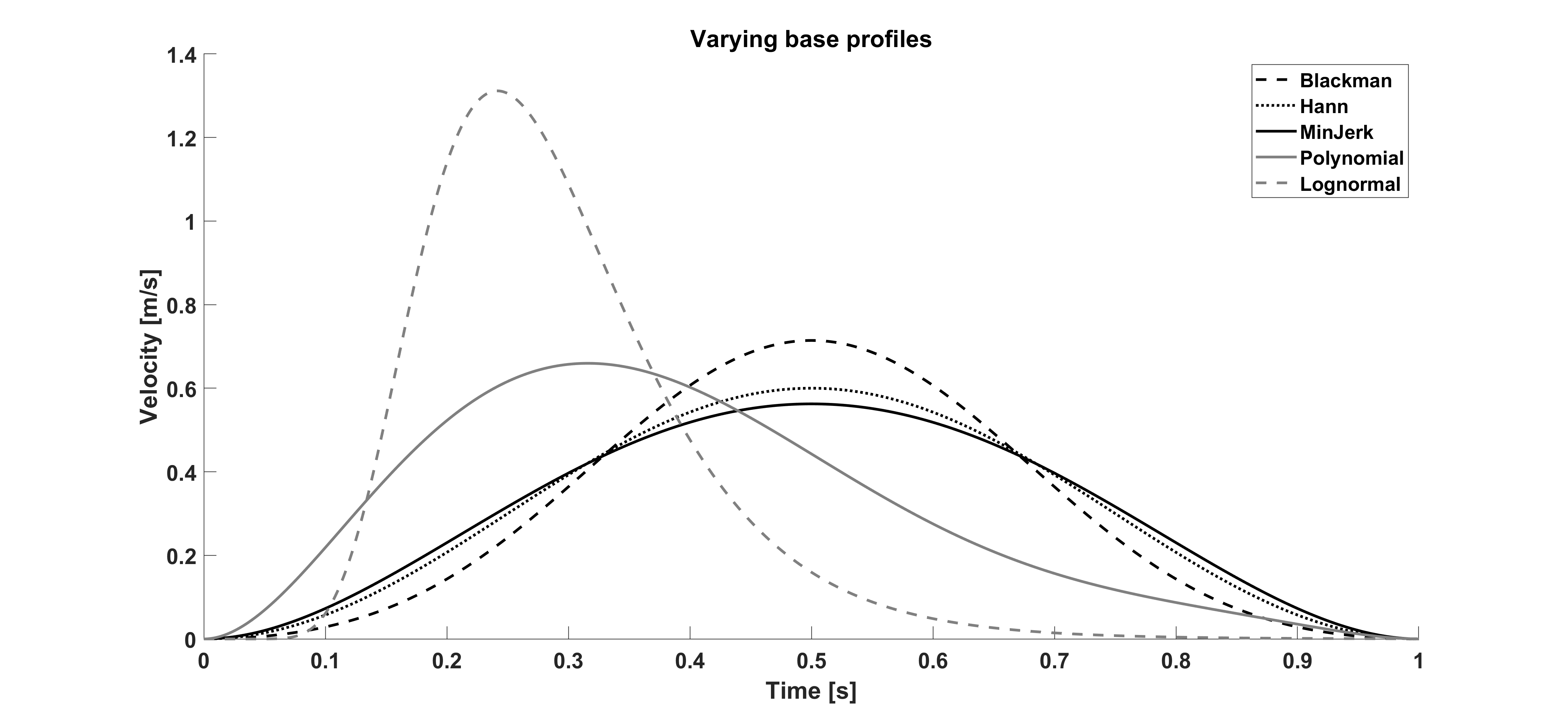


**Fig. G.1:** Varying base profiles used to assess the monotonicity to the Sub-movements Simulation.

For each profile, the sub-movement simulation was performed, and then the derivative of the change in metric for increasing delays was extracted. The percentage change was estimated as follows:

$$change=\frac{\delta metric_{i}- \delta metric_{i-1}}{\delta metric_{max}-\delta metric_{min}}*100$$

where $\delta metric_{i}= metric_{i+1}- metric_{i}$ where $i$ denotes the lag *Ks*. The minimum and maximum values of the change for each number of submovements used (2, 3, and 4) can be seen in Tables AF 1.G 1, 2, and 3.

**Table G.1 Percent change in metrics with increasing delay between 2 sub-movements**

|  | **Blackman**  [min max]  (%) | **Hann**  [min max]  (%) | **Min Jerk**  [min max]  (%) | **Polynomial**  [min max]  (%) | **Lognormal**  [min max]  (%) |
| --- | --- | --- | --- | --- | --- |
| **NOS*** | [-57.1 42.9] | [-50 50] | [-50 50] | [-50 50] | [-50 50] |
| **SM** | [-24.6 75.4] | [-23.2 76.9] | [-24.4 75.6] | [-46.5 53.5] | [-10.3 89.7] |
| **MAPR** | [-78.8 21.2] | [-76.2 23.8] | [-82.5 17.5] | [-59.6 40.4] | [-56.4 43.7] |
| **VAL*** | [28.2 128.2] | [28 128] | [28 128] | [28.3 128.3] | [50.4 150.4] |
| **Peaks*** | [0 100] | [-49.4 50.6] | [-33.3 66.7] | [0 100] | [-50 50] |
| **IPV** | [-100 0] | [-75.4 24.6] | [-85.7 14.3] | [-100 0] | [-83.3 16.7] |
| **DSJt*** | [-25.3 74.7] | [-12.2 87.9] | [-33.3 66.7] | [-11.3 88.7] | [-1.7 98.3] |
| **LDSJT*** | [-32.9 67.1] | [-19.5 80.6] | [-23.9 76.2] | [-9.4 90.6] | [-51.7 48.3] |
| **DSJb*** | [-6.6 93.4] | [-13.7 86.3] | [-50.6 49.4] | [-38.8 61.3] | [0 100] |
| **LDSJb*** | [-18.4 81.6] | [-4.9 95.1] | [-22.5 77.5] | [-22.9 77.2] | [-42.5 57.5] |
| **CM** | [-90.8 9.2] | [-96.4 3.6] | [-98.3 1.7] | [-67.4 32.6] | [-38.2 61.9] |
| **SPMR** | [-55.8 44.2] | [-43.7 56.3] | [-44.7 55.3] | [-52 48.1] | [-50.5 49.5] |
| **SPM** | [-100 0] | [-74.5 25.5] | [-71.9 28.1] | [-53.1 46.9] | [-82.2 17.8] |
| **SPAL** | [-74.2 25.8] | [-76.4 23.6] | [-78.8 21.2] | [-77 23.1] | [-65.6 34.4] |
| **SPARC** | [-85.3 14.7] | [-87.3 12.7] | [-83.7 16.3] | [-90.8 9.2] | [-87.3 12.7] |

**Table G.2 Percent change in metrics with increasing delay between 3 sub-movements**

|  | **Blackman**  [min max]  (%) | **Hann**  [min max]  (%) | **Min Jerk**  [min max]  (%) | **Polynomial**  [min max]  (%) | **Lognormal**  [min max]  (%) |
| --- | --- | --- | --- | --- | --- |
| **NOS*** | [-40 60] | [-42.9 57.1] | [-50 50] | [-60 40] | [-66.7 33.3] |
| **SM** | [-38.3 61.7] | [-37.9 62.1] | [-39.7 60.3] | [-46.3 53.8] | [-19.1 80.9] |
| **MAPR** | [-76.5 23.5] | [-75.2 24.8] | [-82.7 17.3] | [-66.7 33.3] | [-41.9 58.1] |
| **VAL*** | [11.7 111.7] | [11.6 111.6] | [11.5 111.5] | [11.9 111.9] | [41.4 141.4] |
| **Peaks*** | [0 100] | [-49.7 50.3] | [-40 60] | [0 100] | [-50 50] |
| **IPV** | [-100 0] | [-77.4 22.6] | [-85.7 14.3] | [-100 0] | [-88.2 11.8] |
| **DSJt*** | [-16.8 83.2] | [-9.9 90.1] | [-34.7 65.3] | [-10.9 89.1] | [-0.2 99.8] |
| **LDSJT*** | [-31.9 68.1] | [-21.9 78.1] | [-41.9 58.1] | [-14.6 85.4] | [-42.2 57.8] |
| **DSJb*** | [-1.9 98.1] | [-14.9 85.1] | [-52.6 47.4] | [-39.1 60.9] | [0 100] |
| **LDSJb*** | [-19.7 80.3] | [-12.2 87.8] | [-23.6 76.4] | [-22.9 77.1] | [-30.3 69.7] |
| **CM** | [-88.5 11.5] | [-94.2 5.8] | [-95.9 4.1] | [-54.4 45.6] | [-32.7 67.3] |
| **SPMR** | [-50.6 49.4] | [-46.9 53.1] | [-47.4 52.6] | [-49.5 50.6] | [-48.8 51.3] |
| **SPM** | [-17.8 82.2] | [-29 71] | [-34.2 65.8] | [-15.7 84.3] | [-23.2 76.9] |
| **SPAL** | [-68 32] | [-67.8 32.2] | [-67.6 32.4] | [-68.2 31.9] | [-89.6 10.4] |
| **SPARC** | [-72.2 27.8] | [-77.7 22.3] | [-84.2 15.8] | [-83.7 16.3] | [-86.3 13.7] |

**Table G.3 Percent change in metrics with increasing delay between 4 sub-movements**

|  | **Blackman**  [min max]  (%) | **Hann**  [min max]  (%) | **Min Jerk**  [min max]  (%) | **Polynomial**  [min max]  (%) | **Lognormal**  [min max]  (%) |
| --- | --- | --- | --- | --- | --- |
| **NOS*** | [-50 50] | [-50 50] | [-50 50] | [-66.7 33.3] | [-50 50] |
| **SM** | [-43.9 56.1] | [-47.3 52.7] | [-48.6 51.4] | [-48.5 51.5] | [-28 72] |
| **MAPR** | [-76.6 23.4] | [-74 26] | [-79.9 20.1] | [-68.8 31.2] | [-38.1 61.9] |
| **VAL*** | [7.7 107.7] | [7.4 107.4] | [7.4 107.4] | [7.9 107.9] | [6.1 106.1] |
| **Peaks*** | [-48.9 51.1] | [-49.8 50.2] | [-42.9 57.1] | [0 100] | [-50 50] |
| **IPV** | [-75.8 24.2] | [-75.9 24.1] | [-88.2 11.8] | [-100 0] | [-91 9] |
| **DSJt*** | [-11.6 88.4] | [-8 92] | [-35.4 64.6] | [-10.7 89.3] | [-0.1 99.9] |
| **LDSJT*** | [-32.5 67.6] | [-23.6 76.4] | [-52 48] | [-20.3 79.7] | [-39 61] |
| **DSJb*** | [-0.7 99.3] | [-15.5 84.5] | [-53.4 46.6] | [-39.3 60.7] | [0 100] |
| **LDSJb*** | [-20.6 79.4] | [-14.2 85.8] | [-43.6 56.4] | [-21.7 78.4] | [-27.9 72.1] |
| **CM** | [-85.7 14.3] | [-92 8] | [-93.9 6.1] | [-45.7 54.3] | [-28.6 71.5] |
| **SPMR** | [-50.8 49.2] | [-50.8 49.2] | [-51 49] | [-50 50] | [-52.8 47.2] |
| **SPM** | [-28.7 71.3] | [-35.7 64.3] | [-42.4 57.6] | [-18.3 81.7] | [-38 62.1] |
| **SPAL** | [-69 31] | [-66.8 33.2] | [-66.9 33.1] | [-70.3 29.7] | [-96.5 3.5] |
| **SPARC** | [-82.2 17.8] | [-81.3 18.7] | [-85.6 14.4] | [-88.4 11.6] | [-80.7 19.3] |

We see from the three tables that only *VAL** is truly non-monotonic. It was found that the monotonicity of metrics depended on the design of the base velocity profile. For *SPARC*, this could be due to the discontinuity in selecting the cut off frequency [24]. Only *VAL**, *SPMR*, and *SPARC* showed less or were not dependent on the underlying base profile. Moreover, we see that *SPAL* and *SPARC* show a monotonic change when the steps over which the derivative of the simulation was 0.06 s or greater.

# REFERENCES

1. Flash T, Hogan N. The coordination of arm movements: An experimentally confirmed mathematical model. J Neurosci. 1985;5:1688–703.

2. Hughes CML, Mäueler B, Tepper H, Seegelke C. Interlimb coordination during a cooperative bimanual object manipulation task. Laterality. 2013;18:693–709.

3. Rohrer B, Hogan N. Avoiding spurious submovement decompositions II: A scattershot algorithm. Biol Cybern. 2006;94:409–14.

4. Rohrer B, Fasoli S, Krebs HI, Hughes R, Volpe B, Frontera WR, et al. Movement Smoothness Changes during Stroke Recovery. J Neurosci. Society for Neuroscience; 2002;22:8297–304.

5. Mazzoleni S, Filippi M, Carrozza MC, Posteraro F, Puzzolante L, Falchi E. Robot-aided therapy on the upper limb of subacute and chronic stroke patients: A biomechanical approach. Proc 2011 IEEE Int Conf Rehabil Robot. 2011. p. 1–6.

6. Beppu H, Suda M, Tanaka R. Analysis of cerebellar motor disorders by visually guided elbow tracking movement. Brain. 1984;107:787–809.

7. Balasubramanian S, Melendez-Calderon A, Burdet E. A robust and sensitive metric for quantifying movement smoothness. IEEE Trans Biomed Eng. 2012;59:2126–36.

8. Krebs HI, Volpe BT, Palazzo J, Rohrer B, Ferraro M, Fasoli S, et al. Robot aided neuro-rehabilitation in stroke: Interim results on follow-up of 76 patients and on movement indices. Integr Assist Technol Inf Age. IOS Press; 2001. p. 45–59.

9. Brooks VB. Introductory lecture to session III: some examples of programmed limb movements. Brain Res. 1974;71:299–308.

10. Kahn LE, Zygman ML, Rymer WZ, Reinkensmeyer DJ. Robot-assisted reaching exercise promotes arm movement recovery in chronic hemiparetic stroke: A randomized controlled pilot study. J Neuroeng Rehabil. 2006;3.

11. Abdul Rahman H, Khor KX, Yeong CF, Su ELM, Narayanan ALT. The potential of iRest in measuring the hand function performance of stroke patients. Biomed Mater Eng. 2017;28:105–16.

12. Pila O, Duret C, Laborne F, Gracies J, Bayle N, Hutin E. Pattern of improvement in upper limb pointing task kinematics after a 3-month training program with robotic assistance in stroke. J Neuroeng Rehabil. 2017;14:105.

13. Duff M, Chen Y, Attygalle S, Herman J, Sundaram H, Qian G, et al. An adaptive mixed reality training system for stroke rehabilitation. IEEE Trans Neural Syst Rehabil Eng. 2010;18:531–41.

14. Bigoni M, Baudo S, Cimolin V, Cau N, Galli M, Pianta L, et al. Does kinematics add meaningful information to clinical assessment in post-stroke upper limb rehabilitation? A case report. J Phys Ther Sci. 2016;28:2408–13.

15. Laczko J, Scheidt RA, Simo LS, Piovesan D. Inter-Joint Coordination Deficits Revealed in the Decomposition of Endpoint Jerk During Goal-Directed Arm Movement After Stroke. IEEE Trans Neural Syst Rehabil Eng. 2017;25:798–810.

16. Young RP, Marteniuk RG. Acquisition of a multi-articular kicking task: Jerk analysis demonstrates movements do not become smoother with learning. Hum Mov Sci. 1997;16:677–701.

17. Adamovich S V., Fluet GG, Merians AS, Mathai A, Qiu Q. Incorporating haptic effects into three-dimensional virtual environments to train the hemiparetic upper extremity. IEEE Trans Neural Syst Rehabil Eng. 2009;17:512–20.

18. Teulings HL, Contreras-Vidal JL, Stelmach GE, Adler CH. Parkinsonism reduces coordination of fingers, wrist, and arm in fine motor control. Exp Neurol. 1997;146:159–70.

19. van Kordelaar J, van Wegen EEH, Kwakkel G. Impact of Time on Quality of Motor Control of the Paretic Upper Limb After Stroke. Arch Phys Med Rehabil. 2014;95:338–44.

20. Marini F, Hughes CML, Squeri V, Doglio L, Moretti P, Morasso P, et al. Robotic wrist training after stroke: Adaptive modulation of assistance in pediatric rehabilitation. Rob Auton Syst. 2017;91:169–78.

21. Repnik E, Puh U, Goljar N, Munih M, Mihelj M. Using Inertial Measurement Units and Electromyography to Quantify Movement during Action Research Arm Test Execution. Sensors. 2018;18:2767.

22. Strohrmann C, Labruyère R, Gerber CN, van Hedel HJ, Arnrich B, Tröster G. Monitoring motor capacity changes of children during rehabilitation using body-worn sensors. J NeuroEngineering Rehabil. 2013;10:83.

23. Balasubramanian S, Wei R, Herman R, He J. Robot-measured performance metrics in stroke rehabilitation. Proc 2009 ICME Int Conf Complex Med Eng C 2009. 2009.

24. Balasubramanian S, Melendez-Calderon A, Roby-Brami A, Burdet E. On the analysis of movement smoothness. J Neuroeng Rehabil. Journal of NeuroEngineering and Rehabilitation; 2015;12:1–11.

25. Kostic M, Popovic M. The modified drawing test for assessment of arm movement quality. J Autom Control. 2013;21:49–53.

26. Simonsen D, Popovic MB, Spaich EG, Andersen OK. Design and test of a Microsoft Kinect-based system for delivering adaptive visual feedback to stroke patients during training of upper limb movement. Med Biol Eng Comput. 2017;55:1927–35.

27. Liebermann DG, Levin MF, McIntyre J, Weiss PL, Berman S. Arm path fragmentation and spatiotemporal features of hand reaching in healthy subjects and stroke patients. Proc 2010 Annu Int Conf IEEE Eng Med Biol. IEEE; 2010. p. 5242–5.

28. Xu C, Li S, Wang K, Hou Z, Yu N. Quantitative assessment of paretic limb dexterity and interlimb coordination during bilateral arm rehabilitation training. 2017 Int Conf Rehabil Robot. IEEE; 2017. p. 634–9.

29. Dipietro L, Krebs HI, Volpe BT, Stein J, Bever C, Mernoff ST, et al. Learning, Not Adaptation, Characterizes Stroke Motor Recovery: Evidence From Kinematic Changes Induced by Robot-Assisted Therapy in Trained and Untrained Task in the Same Workspace. IEEE Trans Neural Syst Rehabil Eng. 2012;20:48–57.

30. Shin J-H, Park G, Cho DY. Cognitive-Motor Interference on Upper Extremity Motor Performance in a Robot-Assisted Planar Reaching Task Among Patients With Stroke. Arch Phys Med Rehabil. 2017;98:730–7.

31. Colombo R, Cusmano I, Sterpi I, Mazzone A, Delconte C, Pisano F. Test–Retest Reliability of Robotic Assessment Measures for the Evaluation of Upper Limb Recovery. IEEE Trans Neural Syst Rehabil Eng. 2014;22:1020–9.

32. Colombo R, Pisano F, Delconte C, Mazzone A, Grioni G, Castagna M, et al. Comparison of exercise training effect with different robotic devices for upper limb rehabilitation: a retrospective study. Eur J Phys Rehabil Med. 2017;53:240–8.

33. Mazzoleni S, Sale P, Tiboni M, Franceschini M, Carrozza MC, Posteraro F. Upper Limb Robot-Assisted Therapy in Chronic and Subacute Stroke Patients. Am J Phys Med Rehabil. 2013;92:e26-37.

34. Giacobbe V, Krebs HI, Volpe BT, Pascual-Leone A, Rykman A, Zeiarati G, et al. Transcranial direct current stimulation (tDCS) and robotic practice in chronic stroke: The dimension of timing. Krebs HI, editor. NeuroRehabilitation. IOS Press; 2013;33:49–56.

35. Gilliaux M, Lejeune T, Detrembleur C, Sapin J, Dehez B, Stoquart G. A robotic device as a sensitive quantitative tool to assess upper limb impairments in stroke patients: A preliminary prospective cohort study. J Rehabil Med. 2012;44:210–7.

36. Christopher SM, Johnson MJ. Task-oriented robot-assisted stroke therapy of paretic limb improves control in a unilateral and bilateral functional drink task: A case study. 2014 36th Annu Int Conf IEEE Eng Med Biol Soc. IEEE; 2014. p. 1194–7.

37. Yoo DH, Kim SY. Effects of upper limb robot-assisted therapy in the rehabilitation of stroke patients. J Phys Ther Sci. 2015;27:677–9.

38. Adams RJ, Lichter MD, Krepkovich ET, Ellington A, White M, Diamond PT. Assessing Upper Extremity Motor Function in Practice of Virtual Activities of Daily Living. IEEE Trans Neural Syst Rehabil Eng. 2015;23:287–96.

39. Duret C, Courtial O, Grosmaire AG. Kinematic measures for upper limb motor assessment during robot-mediated training in patients with severe sub-acute stroke. Restor Neurol Neurosci. 2016;34:237–45.

40. Mazzoleni S, Buono L, Dario P, Posteraro F. Upper limb robot-assisted therapy in subacute and chronic stroke patients: Preliminary results on initial exposure based on kinematic measures. Proc IEEE RAS EMBS Int Conf Biomed Robot Biomechatronics. 2014;265–9.

41. Daly JJ, Hogan N, Perepezko EM, Krebs HI, Rogers JM, Goyal KS, et al. Response to upper-limb robotics and functional neuromuscular. J Rehabil Res Dev. 2005;42:723.

42. Celik O, O’Malley MK, Boake C, Levin HS, Yozbatiran N, Reistetter TA. Normalized movement quality measures for therapeutic robots strongly correlate with clinical motor impairment measures. IEEE Trans Neural Syst Rehabil Eng. 2010;18.

43. Kahn LE, Zygman ML, Rymer WZ, Reinkensmeyer DJ. Effect of robot-assisted and unassisted exercise on functional reaching in chronic hemiparesis. 2001 Conf Proc 23rd Annu Int Conf IEEE Eng Med Biol Soc. 2001;2:1344–7.

44. Kamper DG, McKenna-Cole AN, Kahn LE, Reinkensmeyer DJ. Alterations in reaching after stroke and their relation to movement direction and impairment severity. Arch Phys Med Rehabil. W B Saunders; 2002;83:702–7.

45. Chang JJ, Tung WL, Wu WL, Su FC. Effect of bilateral reaching on affected arm motor control in stroke – with and without loading on unaffected arm. Disabil Rehabil. 2006;28:1507–16.

46. Casadio M, Giannoni P, Morasso P, Sanguineti V. A proof of concept study for the integration of robot therapy with physiotherapy in the treatment of stroke patients. Clin Rehabil. 2009;23:217–28.

47. Senesac CR, Davis S, Richards L. Generalization of a modified form of repetitive rhythmic bilateral training in stroke. Hum Mov Sci. 2010;29:137–48.

48. Murphy MA, Willén C, Sunnerhagen KS. Kinematic Variables Quantifying Upper-Extremity Performance After Stroke During Reaching and Drinking From a Glass. Neurorehabil Neural Repair. 2011;25:71–80.

49. Bustrén E-L, Sunnerhagen KS, Alt Murphy M. Movement Kinematics of the Ipsilesional Upper Extremity in Persons With Moderate or Mild Stroke. Neurorehabil Neural Repair. 2017;31:376–86.

50. Bermúdez i Badia S, Cameirão MS. The Neurorehabilitation Training Toolkit (NTT): A novel worldwide accessible motor training approach for at-home rehabilitation after stroke. Stroke Res Treat. 2012;2012.

51. Levin MF, Magdalon EC, Michaelsen SM, Quevedo AAF. Quality of Grasping and the Role of Haptics in a 3-D Immersive Virtual Reality Environment in Individuals With Stroke. IEEE Trans Neural Syst Rehabil Eng. 2015;23:1047–55.

52. Tropea P, Cesqui B, Monaco V, Aliboni S, Posteraro F, Micera S. Effects of the Alternate Combination of “Error-Enhancing” and “Active Assistive” Robot-Mediated Treatments on Stroke Patients. IEEE J Transl Eng Heal Med. 2013;1:2100109–2100109.

53. Colombo R, Sterpi I, Mazzone A, Delconte C, Minuco G, Pisano F. Measuring changes of movement dynamics during robot-aided neurorehabilitation of stroke patients. IEEE Trans Neural Syst Rehabil Eng. 2010;18.

54. Chalmers N, Glasgow J, Scott S. Dynamic Time Warping as a spatial assessment of sensorimotor impairment resulting from stroke. 2011 Annu Int Conf IEEE Eng Med Biol Soc. 2011. p. 8235–8.

55. Frisoli A, Sotgiu E, Procopio C, Bergamasco M, Chisari C, Lamola G, et al. Training and assessment of upper limb motor function with a robotic exoskeleton after stroke. Proc IEEE RAS EMBS Int Conf Biomed Robot Biomechatronics. 2012.

56. Alt Murphy M, Willén C, Sunnerhagen KS. Movement Kinematics During a Drinking Task Are Associated With the Activity Capacity Level After Stroke. Neurorehabil Neural Repair. M. Alt Murphy, Neuroscience and Physiology, University of Gothenburg, Per Dubbsgatan 14, 3tr, Gothenburg, SE-41345, Sweden; 2012;26:1106–15.

57. Frisoli A, Procopio C, Chisari C, Creatini I, Bonfiglio L, Bergamasco M, et al. Positive effects of robotic exoskeleton training of upper limb reaching movements after stroke. J NeuroEngineering Rehabil. BioMed Central; 2012;9:36.

58. Metrot J, Froger J, Hauret I, Mottet D, Van Dokkum L, Laffont I. Motor recovery of the ipsilesional upper limb in subacute stroke. Arch Phys Med Rehabil. Elsevier; 2013;94:2283–90.

59. van Dokkum L, Hauret I, Mottet D, Froger J, Métrot J, Laffont I. The Contribution of Kinematics in the Assessment of Upper Limb Motor Recovery Early After Stroke. Neurorehabil Neural Repair. 2014;28:4–12.

60. Mohapatra S, Harrington R, Chan E, Dromerick AW, Breceda EY, Harris-Love M. Role of contralesional hemisphere in paretic arm reaching in patients with severe arm paresis due to stroke: A preliminary report. Neurosci Lett. 2016;617:52–8.

61. Park H, Kim S, Winstein CJ, Gordon J, Schweighofer N. Short-Duration and Intensive Training Improves Long-Term Reaching Performance in Individuals With Chronic Stroke. Neurorehabil Neural Repair. 2016;30:551–61.

62. Longhi M, Merlo A, Prati P, Giacobbi M, Mazzoli D. Instrumental indices for upper limb function assessment in stroke patients : a validation study. J Neuroeng Rehabil. Journal of NeuroEngineering and Rehabilitation; 2016;1–11.

63. Gulde P, Hughes CML, Hermsdörfer J. Effects of Stroke on Ipsilesional End-Effector Kinematics in a Multi-Step Activity of Daily Living. Front Hum Neurosci. 2017;11.

64. Hussain N, Alt Murphy M, Sunnerhagen KS. Upper Limb Kinematics in Stroke and Healthy Controls Using Target-to-Target Task in Virtual Reality. Front Neurol. 2018;9:1–9.

65. Hussain N, Sunnerhagen KS, Alt Murphy M. End-point kinematics using virtual reality explaining upper limb impairment and activity capacity in stroke. J Neuroeng Rehabil. Journal of NeuroEngineering and Rehabilitation; 2019;16:1–9.

66. Tomita Y, Mullick AA, Levin MF. Reduced Kinematic Redundancy and Motor Equivalence During Whole-Body Reaching in Individuals With Chronic Stroke. Neurorehabil Neural Repair. 2018;32:175–86.

67. van Dokkum LEH, le Bars E, Mottet D, Bonafé A, Menjot de Champfleur N, Laffont I. Modified Brain Activations of the Nondamaged Hemisphere During Ipsilesional Upper-Limb Movement in Persons With Initial Severe Motor Deficits Poststroke. Neurorehabil Neural Repair. 2018;32:34–45.

68. Schneider S, Schönle PW, Altenmüller E, Münte TF. Using musical instruments to improve motor skill recovery following a stroke. J Neurol. 2007;254:1339–46.

69. Amengual JL, Rojo N, Veciana de las Heras M, Marco-Pallarés J, Grau-Sánchez J, Schneider S, et al. Sensorimotor Plasticity after Music-Supported Therapy in Chronic Stroke Patients Revealed by Transcranial Magnetic Stimulation. PLoS One. 2013;8.

70. Cha Y-J, Yoo E-Y, Jung M-Y, Park S-H, Park J-H, Lee J. Effects of Mental Practice with Action Observation Training on Occupational Performance after Stroke. J Stroke Cerebrovasc Dis. 2015;24:1405–13.

71. Johnson MJ, Wisneski K, Hermsen A, Smith RQ, Walton T, Hingtgen B, et al. Kinematic implications of learned non-use for robotic therapy. Proc 2005 IEEE 9th Int Conf Rehabil Robot. 2005.

72. Wu C, Trombly CA, Lin K, Tickle-Degnen L. A kinematic study of contextual effects on reaching performance in persons with and without stroke: Influences of object availability. Arch Phys Med Rehabil. W B Saunders; 2000;81:aapmr0810095.

73. Chang J-J, Yang Y-S, Wu W-L, Guo L-Y, Su F-C. The constructs of kinematic measures for reaching performance in stroke patients. J Med Biol Eng. 2008;28:65–70.

74. Massie CL, Malcolm MP. Instructions emphasizing speed improves hemiparetic arm kinematics during reaching in stroke. NeuroRehabilitation. 2012;30:341–50.

75. Lee DH, Kim WJ, Oh JS, Chang M. Taping of the elbow extensor muscle in chronic stroke patients: Comparison between before and after three-dimensional motion analysis. J Phys Ther Sci. 2015;27.

76. Casadio M, Morasso P, Noriaki Ide A, Sanguineti V, Giannoni P. Measuring functional recovery of hemiparetic subjects during gentle robot therapy. Meas J Int Meas Confed. Elsevier Ltd; 2009;42:1176–87.

77. Park J-H, Yoo E, Chung B, Jung M. Effects of vocalization on elbow motion during reaching in persons with hemiparetic stroke. NeuroRehabilitation. 2009;25:123–8.

78. Tsao C-C, Mirbagheri MM. Upper limb impairments associated with spasticity in neurological disorders. J Neuroeng Rehabil. 2007;4:45.

79. Alt Murphy M, Murphy S, Persson HC, Bergström UB, Sunnerhagen KS. Kinematic analysis using 3D motion capture of drinking task in people with and without upper-extremity impairments. J Vis Exp. 2018;2018.

80. Trombly CA, Wu C-Y. Effect of Rehabilitation Tasks on Organization of Movement After Stroke. Am J Occup Ther. 1999;53:333–44.

81. Wu C-Y, Chou S-H, Chen C-L, Kuo M-Y, Lu T-W, Fu Y-C. Kinematic analysis of a functional and sequential bimanual task in patients with left hemiparesis: intra-limb and interlimb coordination. Disabil Rehabil. 2009;31:958–66.

82. Coqueiro PR, De Freitas SMSF, Silva CMAE, Alouche SR. Effects of direction and index of difficulty on aiming movements after stroke. Behav Neurol. 2014;2014.

83. Singh T, Perry CM, Fritz SL, Fridriksson J, Herter TM. Eye Movements Interfere With Limb Motor Control in Stroke Survivors. Neurorehabil Neural Repair. 2018;32:724–34.

84. Schweighofer N, Wang C, Mottet D, Laffont I, Bakhti K, Reinkensmeyer DJ, et al. Dissociating motor learning from recovery in exoskeleton training post-stroke. J Neuroeng Rehabil. Journal of NeuroEngineering and Rehabilitation; 2018;15:89.

85. Richards L, Senesac C, McGuirk T, Woodbury M, Howland D, Davis S, et al. Response to Intensive Upper Extremity Therapy by Individuals with Ataxia from Stroke. Top Stroke Rehabil. 2008;15:262–71.

86. Bensmail D, Robertson JVG, Fermanian C, Roby-Brami A. Botulinum toxin to treat upper-limb spasticity in hemiparetic patients: Analysis of function and kinematics of reaching movements. Neurorehabil Neural Repair. 2010;24.

87. Alt Murphy M, Willén C, Sunnerhagen KS. Responsiveness of Upper Extremity Kinematic Measures and Clinical Improvement During the First Three Months After Stroke. Neurorehabil Neural Repair. 2013;27:844–53.

88. Pila O, Duret C, Gracies J-M, Francisco GE, Bayle N, Hutin É. Evolution of upper limb kinematics four years after subacute robot-assisted rehabilitation in stroke patients. Int J Neurosci. Taylor & Francis; 2018;128:1030–9.

89. Wu C, Yang C, Chuang L, Lin K, Chen H, Chen M, et al. Effect of Therapist-Based Versus Robot-Assisted Bilateral Arm Training on Motor Control, Functional Performance, and Quality of Life After Chronic Stroke: A Clinical Trial. Phys Ther. 2012;92:1006–16.

90. Menegoni F, Milano E, Trotti C, Galli M, Bigoni M, Baudo S, et al. Quantitative evaluation of functional limitation of upper limb movements in subjects affected by ataxia. Eur J Neurol. 2009;16:232–9.

91. Salazar AP, Cimolin V, Schifino GP, Rech KD, Marchese RR, Pagnussat AS. Bi-cephalic transcranial direct current stimulation combined with functional electrical stimulation for upper-limb stroke rehabilitation: A double-blind randomized controlled trial. Ann Phys Rehabil Med. 2020;63:4–11.

92. Schiefelbein ML, Salazar AP, Marchese RR, Rech KD, Schifino GP, Figueiredo CS, et al. Upper-limb movement smoothness after stroke and its relationship with measures of body function/structure and activity – A cross-sectional study. J Neurol Sci. 2019;401:75–8.

93. Wu C, Chuang L, Lin K, Chen H, Tsay P. Randomized Trial of Distributed Constraint-Induced Therapy Versus Bilateral Arm Training for the Rehabilitation of Upper-Limb Motor Control and Function After Stroke. Neurorehabil Neural Repair. 2011;25:130–9.

94. Szczȩsna A, Błaszczyszyn M. Quantitative analysis of arm movement smoothness. AIP Conf Proc. 2017.

95. Merians AS, Fluet GG, Qiu Q, Adamovich S V. Robotically facilitated training of the hemiparetic upper extremity as an integrated functional unit in virtual environments. 2009 Virtual Rehabil Int Conf VR 2009. 2009.

96. Song R, Tong KY, Hu XL. Evaluation of Velocity-Dependent Performance of the Spastic Elbow During Voluntary Movements. Arch Phys Med Rehabil. 2008;89:1140–5.

97. Mazzoleni S, Tran V-D, Dario P, Posteraro F. Wrist Robot-Assisted Rehabilitation Treatment in Subacute and Chronic Stroke Patients: From Distal-to-Proximal Motor Recovery. IEEE Trans Neural Syst Rehabil Eng. S. Mazzoleni, Polo sant’Anna Valdera, Scuola Superiore sant’Anna, BioRobotics Institute, Pontedera, Italy: IEEE; 2018;26:1889–96.

98. Merians AS, Fluet GG, Qiu Q, Saleh S, Lafond I, Davidow A, et al. Robotically facilitated virtual rehabilitation of arm transport integrated with finger movement in persons with hemiparesis. J Neuroeng Rehabil. 2011;8:27.

99. Tavernese E, Paoloni M, Mangone M, Mandic V, Sale P, Franceschini M, et al. Segmental muscle vibration improves reaching movement in patients with chronic stroke. A randomized controlled trial. NeuroRehabilitation. IRCSS San Raffaele, Rome, Italy: IOS Press; 2013;32:591–9.

100. Bartolo M, De Nunzio AM, Sebastiano F, Spicciato F, Tortola P, Nilsson J, et al. Arm weight support training improves functional motor outcome and movement smoothness after stroke. Funct Neurol. 2014;29:15–21.

101. Buma FE, van Kordelaar J, Raemaekers M, van Wegen EEH, Ramsey NF, Kwakkel G. Brain activation is related to smoothness of upper limb movements after stroke. Exp Brain Res. 2016;234:2077–89.

102. Kato N, Tanaka T, Sugihara S, Shimizu K, Kudo N. A study of the effect of visual depth information on upper limb movement by use of measurement of smoothness. J Phys Ther Sci. 2016;28.

103. Scano A, Chiavenna A, Malosio M, Molinari Tosatti L, Molteni F. Muscle Synergies-Based Characterization and Clustering of Poststroke Patients in Reaching Movements. Front Bioeng Biotechnol. 2017;5.

104. Osumi M, Sumitani M, Otake Y, Morioka S. A “matched” sensory reference can guide goal-directed movements of the affected hand in central post-stroke sensory ataxia. Exp Brain Res. 2018;236:1263–72.

105. Cusmano I, Colombo R, Sterpi I, Mazzone A, Delconte C, Pisano F. Inter-session reliability of robot-measured parameters for the evaluation of upper limb recovery. Biosyst Biorobotics. 2014;7.

106. Hussain A, Budhota A, Hughes CML, Dailey WD, Vishwanath DA, Kuah CWK, et al. Self-Paced Reaching after Stroke: A Quantitative Assessment of Longitudinal and Directional Sensitivity Using the H-Man Planar Robot for Upper Limb Neurorehabilitation. Front Neurosci. 2016;10.

107. Budhota A, Hussain A, Hughes C, Hansen C, Kager S, Vishwanath DA, et al. Role of EMG as a complementary tool for assessment of motor impairment. 2016 6th IEEE Int Conf Biomed Robot Biomechatronics. IEEE; 2016. p. 692–7.

108. Hussain A, Dailey W, Hughes C, Budhota A, Gamage WGKC, Vishwanath DA, et al. Quantitative motor assessment of upperlimb after unilateral stroke: A preliminary feasibility study with H-Man, a planar robot. IEEE Int Conf Rehabil Robot. 2015.

109. Leconte P, Orban de Xivry J-J, Stoquart G, Lejeune T, Ronsse R. Rhythmic arm movements are less affected than discrete ones after a stroke. Exp Brain Res. 2016;234:1403–17.

110. Popović MD, Kostić MD, Rodić SZ, Konstantinović LM. Feedback-Mediated Upper Extremities Exercise: Increasing Patient Motivation in Poststroke Rehabilitation. Biomed Res Int. 2014;2014:1–11.
